# Deep learning for multi-type infectious keratitis diagnosis: A nationwide, cross-sectional, multicenter study

**DOI:** 10.1038/s41746-024-01174-w

**Published:** 2024-07-06

**Authors:** Zhongwen Li, He Xie, Zhouqian Wang, Daoyuan Li, Kuan Chen, Xihang Zong, Wei Qiang, Feng Wen, Zhihong Deng, Limin Chen, Huiping Li, He Dong, Pengcheng Wu, Tao Sun, Yan Cheng, Yanning Yang, Jinsong Xue, Qinxiang Zheng, Jiewei Jiang, Wei Chen

**Affiliations:** 1 Ningbo Key Laboratory of Medical Research on Blinding Eye Diseases, Ningbo Eye Institute, Ningbo Eye Hospital, Wenzhou Medical University, Ningbo, 315000 China; 2 National Clinical Research Center for Ocular Diseases, Eye Hospital, Wenzhou Medical University, Wenzhou, 325027 China; 3 Department of Ophthalmology, The Affiliated Hospital of Guizhou Medical University, Guiyang, 550004 China; 4 Department of Ophthalmology, Cangnan Hospital, Wenzhou Medical University, Wenzhou, 325000 China; 5 Department of Ophthalmology, The Third Xiangya Hospital, Central South University, Changsha, 410013 China; 6 Department of Ophthalmology, The First Affiliated Hospital of Fujian Medical University, Fuzhou, 350000 China; 7 Department of Ophthalmology, People’s Hospital of Ningxia Hui Autonomous Region, Ningxia Medical University, Yinchuan, 750001 China; 8 The Third People’s Hospital of Dalian & Dalian Municipal Eye Hospital, Dalian, 116033 China; 9 Department of Ophthalmology, The Second Hospital of Lanzhou University, Lanzhou, 730030 China; 10 The Affiliated Eye Hospital of Nanchang University, Jiangxi Clinical Research Center for Ophthalmic Disease, Jiangxi Research Institute of Ophthalmology and Visual Science, Jiangxi Provincial Key Laboratory for Ophthalmology, Nanchang, 330006 China; 11 Xi’an No.1 Hospital, Shaanxi Institute of Ophthalmology, Shaanxi Key Laboratory of Ophthalmology, The First Affiliated Hospital of Northwestern University, Xi’an, 710002 China; 12 Department of Ophthalmology, Renmin Hospital of Wuhan University, Wuhan, 430060 China; 13 Affiliated Eye Hospital of Nanjing Medical University, Nanjing, 210029 China; 14 School of Electronic Engineering, Xi’an University of Posts and Telecommunications, Xi’an, 710121 China

**Keywords:** Corneal diseases, Diagnosis

## Abstract

The main cause of corneal blindness worldwide is keratitis, especially the infectious form caused by bacteria, fungi, viruses, and Acanthamoeba. The key to effective management of infectious keratitis hinges on prompt and precise diagnosis. Nevertheless, the current gold standard, such as cultures of corneal scrapings, remains time-consuming and frequently yields false-negative results. Here, using 23,055 slit-lamp images collected from 12 clinical centers nationwide, this study constructed a clinically feasible deep learning system, DeepIK, that could emulate the diagnostic process of a human expert to identify and differentiate bacterial, fungal, viral, amebic, and noninfectious keratitis. DeepIK exhibited remarkable performance in internal, external, and prospective datasets (all areas under the receiver operating characteristic curves > 0.96) and outperformed three other state-of-the-art algorithms (DenseNet121, InceptionResNetV2, and Swin-Transformer). Our study indicates that DeepIK possesses the capability to assist ophthalmologists in accurately and swiftly identifying various infectious keratitis types from slit-lamp images, thereby facilitating timely and targeted treatment.

## Introduction

Corneal blindness, mainly arising from keratitis, particularly infectious keratitis (IK) caused by bacteria, fungi, viruses, and Acanthamoeba, ranks fifth among the leading causes of visual impairment on a global scale^1–3^. Based on a recent report by the World Health Organization, IK affects around 6 million individuals worldwide and contributes to an annual count of 1.5 to 2.0 million cases of monocular blindness^3,4^. As an urgent condition in ophthalmology, IK usually needs immediate treatment^2–4^. Otherwise, the infection could go through the cornea and disseminate to various ocular structures, resulting in significant visual impairment and potentially grave complications including corneal perforation and endophthalmitis^5,6^.

Prompt detection of the cause of IK is the premise of providing targeted therapy for reducing vision loss and preventing severe complications^2,5^. However, due to overlapping inflammatory features, even ophthalmologists have relatively poor performance in the identification of pathogenic microorganisms of IK^7–9^. For example, in distinguishing among bacterial, fungal, and amebic keratitis, ophthalmologists can only achieve an accuracy of 73%^9^. In addition, the lack of skilled ophthalmologists, especially in remote and undeveloped areas, further hinders the expeditious determination of the underlying cause of IK^10^. Moreover, although corneal scraping culture remains the gold standard for identifying the etiology of IK caused by bacteria or fungi, culture results are not immediately available (often requiring 48 hours or more) and subsequent identification of microorganisms exists in only 40 to 60% of cases^7,8^. Therefore, developing a method that can accurately and swiftly discern the cause of IK is crucial to helping IK patients reduce their vision loss with timely and effective treatment.

In the past decade, artificial intelligence (AI) techniques have been introduced into the domain of ophthalmology, exhibiting excellent diagnostic performance in identifying conditions such as corneal, retinal, and optic nerve disorders^11–16^. Several studies have utilized deep learning to differentiate between bacterial and fungal keratitis by analyzing slit-lamp images^15,17,18^. However, their proposed models may not be applicable in clinical practice as the major categories of keratitis in the real world also include viral, amebic, and noninfectious keratitis in addition to bacterial and fungal keratitis. Notably, the rise in global usage of contact lenses has resulted in an escalating incidence of amebic keratitis^19^. Precise classification of these categories is critically important as they vary substantially in biological behavior and treatment^2,5^.

In our previous study, an AI system had been designed for the screening of patients with keratitis^20^. To further discern the cause of keratitis, this study developed a customized deep-learning system called DeepIK. This system could mimic the diagnostic process of a human expert in identifying bacterial, fungal, viral, amebic, and noninfectious keratitis from slit-lamp images. Besides, this study evaluated the effectiveness of DeepIK using the datasets retrospectively collected from 12 clinical centers nationwide and the dataset prospectively obtained from the Eye Hospital of Wenzhou Medical University (EHWMU). Furthermore, the performance of DeepIK was compared against ophthalmologists at various levels of expertise.

## Results

### Image data characteristics

After removing 1452 poor-quality images and 3624 images without sufficient diagnostic certainty, datasets comprising 23,055 qualified images were utilized to construct and evaluate a deep learning system. The included images were derived from 10,369 patients across 12 independent clinical centers spanning the entire country. Specifically, the datasets consisted of 3394 images of bacterial keratitis, 4328 images of fungal keratitis, 8224 images of viral keratitis, 446 images of amebic keratitis, and 6663 images of noninfectious keratitis. The patient cohort had a mean age of 53.6 years (with a range of 0.25 to 100 years) and represented 41.8% of women or girls. Comprehensive details regarding the development and external test datasets retrospectively collected from 12 clinical centers nationwide and the dataset prospectively collected from EHWMU are summarized in Table 1. The demographic characteristics of patients from each clinical center are presented in Supplementary Table 1.

**Table 1:** Summary of the development dataset and external and prospective test datasets

Clinical center	Location of center		Bacterial keratitis	Fungal keratitis	Viral keratitis	Amebic keratitis	Noninfectious keratitis	Total
			No of images					
**Development dataset**								
EHWMU	Wenzhou, Zhejiang (Training dataset)		1119	1190	2695	187	2171	7362
EHWMU	Wenzhou, Zhejiang (Validation dataset)		211	267	444	53	473	1448
EHWMU	Wenzhou, Zhejiang (Internal test dataset)		260	271	647	72	532	1782
**External test dataset**								
NEH	Ningbo, Zhejiang		43	131	170	0	132	476
DNPH	Dalian, Liaoning		114	472	245	0	120	951
FAHFMU	Fuzhou, Fujian		50	126	154	9	79	418
SHLU	Lanzhou, Gansu		118	78	39	0	26	261
AEHNU	Nanchang, Jiangxi		51	180	158	30	57	476
AEHNMU	Nanjing, Jiangsu		123	103	27	28	27	308
PHNHAR	Yinchuan, Ningxia		261	130	223	0	262	876
RHWU	Wuhan, Hubei		266	330	374	0	168	1138
XNH	Xi’an, Shaanxi		373	109	288	21	310	1101
TXHCSU	Changsha, Hunan		71	120	428	0	174	793
AHGMU	Guiyang, Guizhou		78	86	82	16	146	408
Total at all centers			1548	1865	2188	104	1501	7206
**Dataset prospectively collected from the real world**								
EHWMU		Wenzhou, Zhejiang	256	735	2250	30	1986	5257

The development dataset is randomly split (7:1.5:1.5) into training, validation, and internal test datasets. The prospective test dataset is independent of the development dataset used to establish the AI system. EHWMU, Eye Hospital of Wenzhou Medical University. NEH, Ningbo Eye Hospital. DNPH, Dalian No.3 People’s Hospital. FAHFMU, First Affiliated Hospital of Fujian Medical University. SHLU, Second Hospital of Lanzhou University. AEHNU, Affiliated Eye Hospital of Nanchang University. AEHNMU, Affiliated Eye Hospital of Nanjing Medical University. PHNHAR, People’s Hospital of Ningxia Hui Autonomous Region. RHWU, Renmin Hospital of Wuhan University. XNH, Xi’an No.1 Hospital. TXHCSU, Third Xiangya Hospital of Central South University. AHGMU, Affiliated Hospital of Guizhou Medical University.

The system was trained and internally evaluated on 10,592 images derived from 6300 patients in the EHWMU, including 1590 images of bacterial keratitis, 1728 of fungal keratitis, 3786 of viral keratitis, 312 of amebic keratitis, and 3176 of noninfectious keratitis. A separate dataset of 7206 images that were obtained from 11 other clinical centers, including 1548 images of bacterial keratitis, 1865 of fungal keratitis, 2188 of viral keratitis, 104 of amebic keratitis, and 1501 of noninfectious keratitis, was utilized for the external testing. The dataset prospectively collected from the EHWMU included 5257 images (256 images of bacterial keratitis, 735 images of fungal keratitis, 2250 images of viral keratitis, 30 images of amebic keratitis, and 1986 images of noninfectious keratitis). The distribution of the 5 types of keratitis across the development dataset and external and prospective test datasets is shown in Supplementary Fig. 1.

In the development dataset, most of the images of infectious keratitis (4146, 55.9%) were diagnosed based on the first criterion (detection of a specific pathogen by laboratory techniques and complete response following definitive therapy), whereas 3270 images (44.1%) were classified based on the second criterion (presence of typical clinical characteristics and complete response following definitive therapy). In the external test dataset, 2573 images (45.1%) of infectious keratitis were diagnosed based on the first criterion, and 3132 images (54.9%) were determined based on the second criterion. In the prospective test dataset, 1809 images (55.3%) of infectious keratitis were labeled based on the first criterion, and 1462 images (44.7%) were determined based on the second criterion.

### Internal evaluation of deep learning models

Four deep learning algorithms, DeepIK, DenseNet121, InceptionResNetV2, and Swin-Transformer, were used to train models for the diagnosis of bacterial, fungal, viral, amebic, and noninfectious keratitis from slit-lamp images. Notably, DeepIK is a customized deep learning algorithm specifically designed to simulate the diagnostic approach of a corneal specialist. Based on the t-distributed stochastic neighbor embedding (t-SNE) technique, DeepIK demonstrated enhanced separability of learned features for each keratitis category compared to DenseNet121, InceptionResNetV2, and Swin-Transformer (Supplementary Fig. 2). The performance of these four algorithms in the internal test dataset is described in Supplementary Fig. 3, revealing that DeepIK is the best algorithm. Details regarding the performance of each algorithm are presented in Supplementary Table 2. The selection of the DenseNet121 convolutional neural network (CNN) architecture as the backbone of DeepIK was based on its superior classification performance compared to InceptionResNetV2 and Swin-Transformer.

The top-performing algorithm, DeepIK, attained area under the receiver operating characteristic curves (AUCs) of 0.949 (95% confidence interval [CI]: 0.937 to 0.960), 0.970 (95% CI: 0.961 to 0.979), 0.955 (95% CI: 0.946 to 0.964), 0.994 (95% CI: 0.988 to 0.999), and 0.979 (95% CI: 0.972 to 0.984) for the classification of bacterial, fungal, viral, amebic, and noninfectious keratitis, respectively. The corresponding accuracies were 91.3% (95% CI: 90.0 to 92.6), 94.1% (95% CI: 93.0 to 95.1), 88.7% (95% CI: 87.3 to 90.2), 98.9% (95% CI: 98.5 to 99.4), and 93.7% (95% CI: 92.5 to 94.8), the corresponding sensitivities were 76.9% (95% CI: 71.8 to 82.0), 79.7% (95% CI: 74.9 to 84.5), 83.5% (95% CI: 80.6 to 86.3), 75.0% (95% CI: 65.0 to 85.0), and 89.3% (95% CI: 86.7 to 91.9), and the corresponding specificities were 93.8% (95% CI: 92.5 to 95.0), 96.6% (95% CI: 95.7 to 97.5), 91.7% (95% CI: 90.1 to 93.3), 99.9% (95% CI: 99.8 to 100), and 95.5% (95% CI: 94.4 to 96.7). In comparison to the reference standard of the internal test dataset, the unweighted Cohen kappa score for DeepIK was 0.773 (95% CI: 0.749 to 0.796).

### External evaluation of deep learning models

**Fig. 1:**
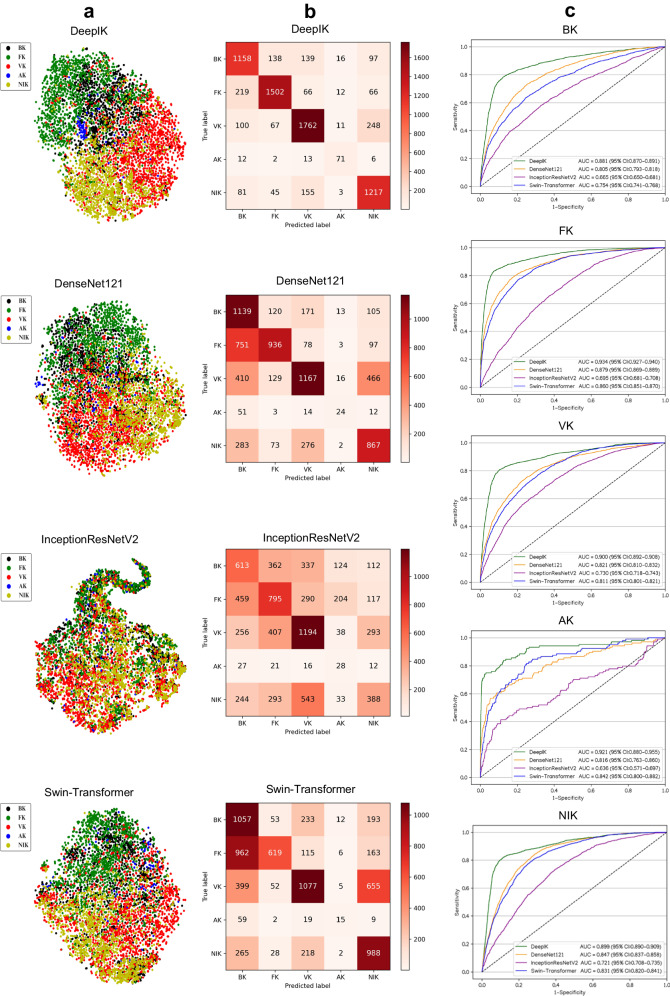
Performance of deep learning algorithms for the diagnosis of bacterial, fungal, viral, amebic, and noninfectious keratitis in external test datasets. **a** Embedding features learned by the deep learning algorithms are projected in two dimensions with t-SNE. The embedding features are represented as dots to show the class distribution. Different colored dot clouds indicate different classes. **b** Confusion matrices showing the accuracies of four deep learning algorithms. **c** Receiver operating characteristic curves of four deep learning algorithms for the classification of bacterial, fungal, viral, amebic, and noninfectious keratitis. BK bacterial keratitis, FK fungal keratitis, VK viral keratitis, AK amebic keratitis, NIK noninfectious keratitis.

In the external test datasets, it was also observed through the t-SNE technique that DeepIK successfully learned more separable features for each category of keratitis compared to DenseNet121, InceptionResNetV2, and Swin-Transformer (Fig. 1a). Similarly, as evident from the confusion matrices (Fig. 1b) and receiver operating characteristic (ROC) curves (Fig. 1c) obtained from the external test datasets, DeepIK exhibited the highest performance in effectively distinguishing between various types of keratitis. For detecting bacterial, fungal, viral, amebic, and noninfectious keratitis, DeepIK attained AUCs of 0.881 (95% CI: 0.870 to 0.891), 0.934 (95% CI: 0.927 to 0.940), 0.900 (95% CI: 0.892 to 0.908), 0.921 (95% CI: 0.880 to 0.955), and 0.899 (95% CI: 0.890 to 0.909), respectively. Detailed information regarding each algorithm’s performance in the external test datasets is provided in Supplementary Table 3. In comparison to the reference standard of the external test datasets, the unweighted Cohen kappa score for DeepIK was 0.724 (95% CI: 0.712 to 0.737).

The algorithms’ performance on each individual external test dataset, is presented in Supplementary Tables 4 to 14. The t-SNE plots, confusion matrices, and ROC curves of the algorithms on each individual external test dataset are presented in Supplementary Figs. 4 to 14.

### Prospective evaluation of deep learning models

**Fig. 2:**
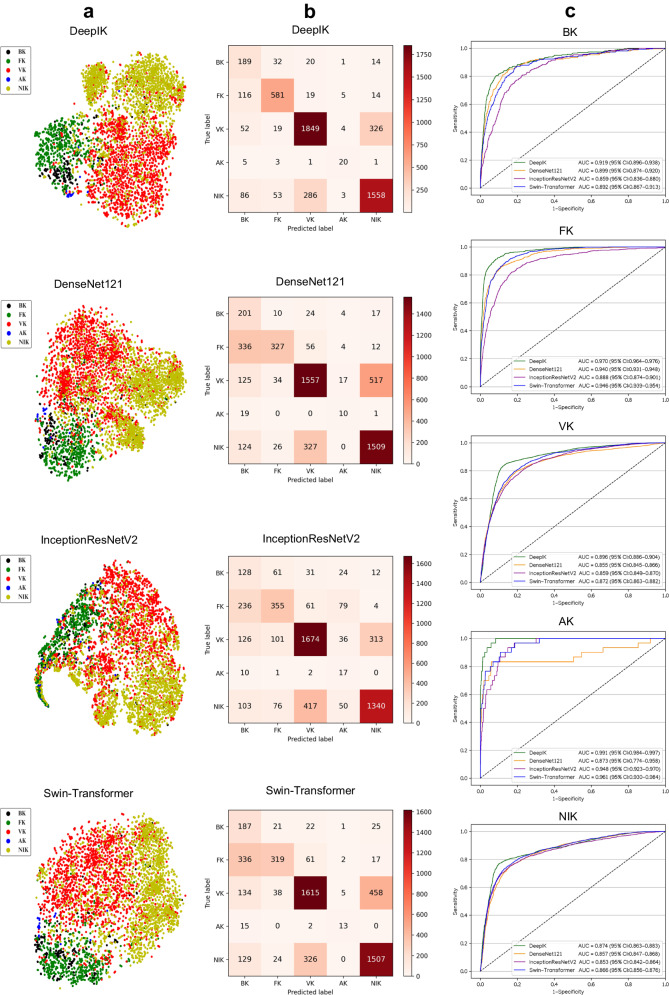
Performance of deep learning algorithms for the diagnosis of bacterial, fungal, viral, amebic, and noninfectious keratitis in a prospective test dataset. **a** Embedding features learned by the deep learning algorithms are projected in two dimensions with t-SNE. The embedding features are represented as dots to show the class distribution. Different colored dot clouds indicate different classes. **b** Confusion matrices showing the accuracies of four deep learning algorithms. **c** Receiver operating characteristic curves of four deep learning algorithms for the classification of bacterial, fungal, viral, amebic, and noninfectious keratitis. BK bacterial keratitis, FK fungal keratitis, VK viral keratitis, AK amebic keratitis, NIK noninfectious keratitis.

The prospective test dataset was collected in Wenzhou, Zhejiang Province, from November 2021 to October 2022. The Performance of DeepIK in this dataset is also better than those of other algorithms (Fig. 2). For the discrimination among bacterial, fungal, viral, amebic, and noninfectious keratitis, DeepIK attained AUCs of 0.919 (95% CI: 0.896 to 0.938), 0.970 (95% CI: 0.964 to 0.976), 0.896 (95% CI: 0.886 to 0.904), 0.991 (95% CI: 0.984 to 0.997), and 0.874 (95% CI: 0.863 to 0.883), respectively. Details regarding each algorithm’s performance in the prospective test dataset are shown in Supplementary Table 15. In comparison to the reference standard of the prospective test datasets, the unweighted Cohen kappa score for DeepIK was 0.696 (95% CI: 0.679 to 0.712).

### Classification errors

A total of 2853 images, constituting 20.0% of the 14,245 images from the internal, external, and prospective test datasets, exhibited discordant outcomes between DeepIK and the reference standard. To be specific, DeepIK misclassified 517 images of bacterial keratitis, 572 images of fungal keratitis, 934 images of viral keratitis, 61 images of amebic keratitis, and 769 images of noninfectious keratitis. Details on classification errors made by DeepIK are depicted in Supplementary Fig. 15. Typical examples of images that were misclassified by DeepIK are provided in Supplementary Fig. 16.

The correlation between the misclassification rates and predicted probabilities of DeepIK is depicted in Supplementary Fig. 17, revealing that as the predicted probabilities declined, both the misclassification rate of each class and the total misclassification rate increased. For all categories, when the predicted probabilities surpass 0.8, the misclassification rates remain below 20%. Conversely, when the probabilities fall below 0.4, the misclassification rate for amebic keratitis is approximately 30%, while the misclassification rates for the remaining four categories exceed 30%. Considering that DeepIK operates as a five-category classification system, it is worth noting that the minimum value of the predicted probabilities generated by DeepIK’s output exceeds 0.2.

### Visual interpretation of DeepIK

To depict visual explanations for DeepIK in identifying bacterial, fungal, viral, amebic, and noninfectious keratitis from slit-lamp images, a heatmap was employed to visually represent the regions that had the greatest impact on DeepIK’s decision-making process. This study observed that the heatmap accentuated areas of pathology in a slit-lamp image, such as corneal infiltration, corneal ulcer, and corneal edema. Representative examples of the heatmaps for bacterial, fungal, viral, amebic, and noninfectious keratitis are shown in Supplementary Fig. 18.

### Comparison between DeepIK and ophthalmologists

A contest dataset of 250 images randomly selected from the external test datasets, including 50 images of bacterial keratitis, 50 images of fungal keratitis, 50 images of viral keratitis, 50 images of amebic keratitis, and 50 images of noninfectious keratitis, was utilized to compare DeepIK’s performance with practicing ophthalmologists in the discrimination among different types of keratitis. For this study, two groups comprising a total of four ophthalmologists were recruited: a junior group consisting of two ophthalmologists possessing 6 to 15 years of clinical experience, and a senior group consisting of two ophthalmologists possessing 16 to 25 years of clinical experience.

**Fig. 3:**
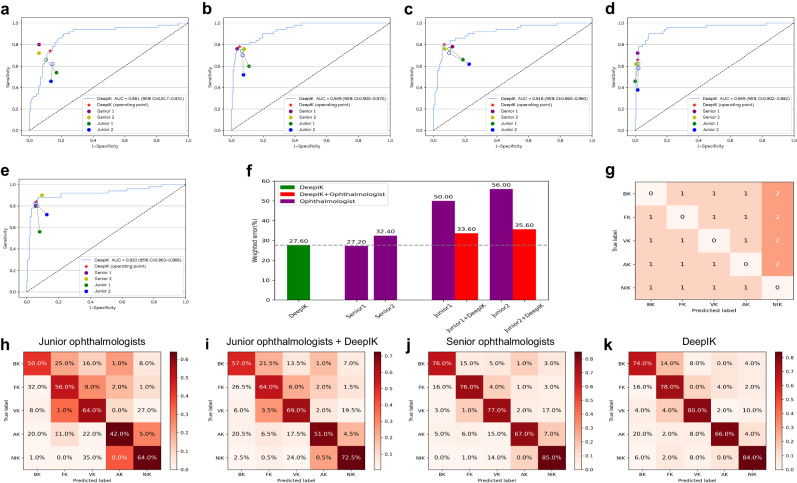
Comparisons of diagnostic performance between DeepIK and practicing ophthalmologists. **a**–**e** The performance of DeepIK and four practicing ophthalmologists (two junior level and two senior level). ROC curves for detecting the different types of keratitis. Filled dots indicate junior and senior ophthalmologists’ performances, while hollow dots indicate the performance of junior ophthalmologists with AI assistance. Dashed lines connected the paired performance metrics of each junior ophthalmologist. **a** ROC curve for diagnosis of bacterial keratitis versus other classes. **b** ROC curve for diagnosis of fungal keratitis versus other classes. **c** ROC curve for diagnosis of viral keratitis versus other classes. **d** ROC curve for diagnosis of amebic keratitis versus other classes. **e** ROC curve for diagnosis of noninfectious keratitis versus other classes. **f** Weighted errors based on penalty scores. **g** Penalty scoring matrix. **h**–**k** Confusion matrices of five-category classification. **h** Confusion matrix of the average diagnostic performance of two junior ophthalmologists. **i** Confusion matrix of the average diagnostic performance of two junior ophthalmologists with DeepIK assistance. **j** Confusion matrix of the average diagnostic performance of two senior ophthalmologists. **k** Confusion matrix of the diagnostic performance of DeepIK.

DeepIK’s overall performance surpassed that of junior ophthalmologists and was on par with senior ophthalmologists (Figs. 3a–e). Detailed information on performance comparison between DeepIK and the ophthalmologists is shown in Supplementary Table 16. To evaluate and compare the performance of DeepIK with that of the ophthalmologists, this study leveraged predicted errors derived from penalty scores (Figs. 3f, g) as a metric. DeepIK exhibited a weighted error of 27.6%, while the ophthalmologists demonstrated a range of weighted errors, spanning from 27.2% to 56.0%, with an average of 41.4% (Fig. 3f).

Comparing the errors made by DeepIK with those made by the ophthalmologists revealed many cases in which DeepIK correctly identified cases whereas all the ophthalmologists did not, and vice versa (Supplementary Table 17). Typical examples that were incorrectly diagnosed by all four ophthalmologists, but correctly diagnosed by DeepIK were shown in Supplementary Fig. 19a. Typical examples that were correctly diagnosed by all four ophthalmologists, but incorrectly diagnosed by DeepIK were shown in Supplementary Fig. 19b. Although the clear patterns among these instances were unable to be determined, the presence of these edge cases suggests potentially complementary roles for DeepIK and the ophthalmologists in reaching accurate conclusions.

To investigate the potential of DeepIK to enhance the diagnostic proficiency of junior ophthalmologists, each participant in this study received diagnostic probability information for each image from DeepIK (Supplementary Fig. 20). Subsequently, they were tasked with making diagnoses, guided by the results generated with the assistance of DeepIK. To mitigate the risk of a memorization bias, the ophthalmologists performed the follow-up diagnostic test with DeepIK assistance four weeks subsequent to the initial examination. The performance of the junior ophthalmologists guided by DeepIK had a great improvement compared to the previous one (Fig. 3). Specifically, with the help of DeepIK, junior ophthalmologist 1 exhibited significantly enhanced accuracies (*P* <0.05) in identifying bacterial, fungal, viral, and noninfectious keratitis and junior ophthalmologist 2 demonstrated significantly improved accuracies (*P* <0.05) in detecting viral and noninfectious keratitis (Supplementary Table 16).

## Discussion

**Fig. 4:**
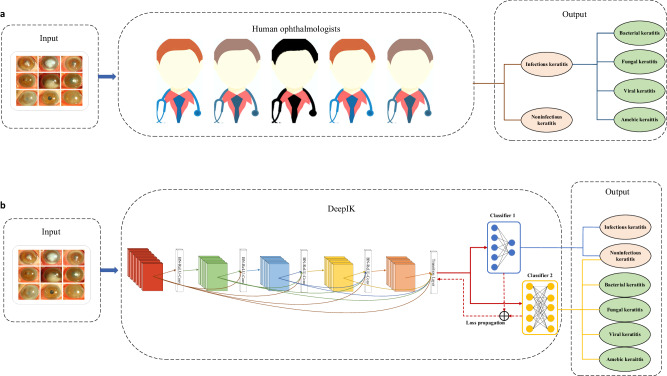
Workflows of human ophthalmologists and DeepIK in identifying the cause of keratitis. **a** The diagnostic pipeline of human ophthalmologists. When human ophthalmologists determine the cause of keratitis based on slit-lamp images, they often first distinguish whether keratitis is infectious or noninfectious. If infectious keratitis is identified, they will further analyze its specific type. **b** The analysis pipeline of DeepIK. The customized algorithm, DeepIK, which includes two classifiers, is employed to imitate the diagnostic process of human ophthalmologists. Classifier 1 is used to differentiate between infectious and noninfectious keratitis. Classifier 2 leveraged the prior knowledge learned from Classifier 1 to further classify keratitis into bacterial, fungal, viral, amebic, and noninfectious keratitis.

In this nationwide study, AI models were constructed for the identification of bacterial, fungal, viral, amebic, and noninfectious keratitis from slit-lamp images and evaluated them on the datasets collected from a variety of commercially available digital slit-lamp cameras from 12 independent clinical centers across the country. Meanwhile, this study developed a customized AI system, DeepIK, for imitating the diagnostic thinking of a human expert in discriminating among different types of keratitis (Fig. 4). Our study found that DeepIK had better performance than other selected deep learning algorithms (DenseNet121, InceptionResNetV2, and Swin-Transformer) in internal, external, and prospective test datasets, demonstrating its robustness and broad generalizability. Besides, Cohen’s unweighted Kappa coefficients demonstrated a substantial level of agreement (0.70–0.77) between the outcomes produced by DeepIK and the reference standard, providing additional evidence of the effectiveness of DeepIK. Moreover, DeepIK took an average of 0.034 seconds to test each image (Supplementary Table 18), indicating that this system could serve as an efficient first-reading tool to assist ophthalmologists in identifying the cause of IK.

To evaluate the level of DeepIK in clinics, this study compared its performance with practicing ophthalmologists of different levels. The results denoted that DeepIK attained comparable performance to the level of senior ophthalmologists and could help junior ophthalmologists improve their diagnostic performance (Fig. 3). This denotes that DeepIK could potentially alleviate the substantial requirement for diagnostic expertise in cases of healthcare system overload or in remote areas where experienced ophthalmologists are scarce.

IK can arise from a diverse range of microorganisms, including bacteria, fungi, viruses, and Acanthamoeba. Precise classification of IK is fairly important because different types of keratitis vary substantially in their biological behavior, their response to therapy, and their clinical prognosis. In real-world clinic settings, achieving a precise and prompt clinical diagnosis of IK proves challenging. This challenge primarily stems from various factors, such as the similarity in clinical features across different causative organisms, a variable and often low culture positivity rate, and prolonged durations for diagnostic testing^3^. However, in clinics, IK is regarded as an urgent condition that necessitates prompt and accurate diagnosis, along with immediate medical interventions. The inability to ascertain the causative agents of IK raises the likelihood of progressive infiltration of the cornea, eventually resulting in suboptimal treatment response and a heightened risk of visual impairment, or in severe cases, blindness. Given its reliable performance (all AUCs over 0.87) and the ability to swiftly provide classification results, DeepIK demonstrates a high potential to assist ophthalmologists in accurately and rapidly distinguishing among various types of IK. This capability could facilitate patients in accessing timely and targeted treatment measures, ultimately enhancing their visual prognosis.

While our system demonstrated robust performance, instances of misclassification persisted. An analysis of the connection between the system’s misclassification rate and its predicted probability was conducted, revealing that lower predicted probabilities correlated with higher misclassification rates. Consequently, images exhibiting low predicted probability values warrant the attention of a doctor. A desirable AI system would aim to minimize false results. Further research is anticipated to delve into the underlying causes of these occurrences and to devise strategies for error reduction.

To enhance the interpretability of DeepIK’s output, this study generated heatmaps to visually depict the areas that the system focused on for making its final decisions. This could assist human experts in the understanding of the justification of DeepIK for discriminating among different types of IK. Heatmaps showed that DeepIK preferentially evaluated corneal lesion regions (e.g., the regions of corneal ulcer, infiltration, and edema). The regions of interest identified by DeepIK aligned with the specific lesions that ophthalmologists closely evaluate during the diagnostic process. The interpretability feature of DeepIK could enhance its applicability in real-world clinical settings.

Recently, Ghosh et al.^15^ proved that deep learning had good performance in discriminating fungal keratitis from bacterial keratitis. They constructed and assessed the system utilizing 2167 slit-lamp images gathered from 194 patients. The system demonstrated an AUC of 0.904, a sensitivity of 0.77, and an F1 score of 0.83. Hung et al.^17^ devised an AI system comprising a segmentation model designed for cornea image cropping and a classification model aimed at distinguishing between bacterial and fungal keratitis. Using 1330 slit-lamp images obtained from 580 patients, their system achieved diagnostic accuracies ranging from 79.6% to 95.9% for bacterial keratitis and from 26.3% to 65.8% for fungal keratitis. Redd et al.^18^ introduced several deep-learning algorithms for discriminating between bacterial and fungal keratitis. Among the algorithms evaluated, MobileNet exhibited the highest performance, achieving an AUC of 0.86 in the single test set comprising 100 images and 0.83 in the multicenter test set consisting of 80 images. Gu et al.^21^ developed a deep learning algorithm, based on 5325 ocular surface images for detecting four common corneal diseases, including infectious keratitis, noninfectious keratitis, corneal dystrophy or degeneration, and corneal and limbal neoplasm. The AUC of their algorithm for each type of corneal disease was over 0.910. Xu et al.^11^ proposed a sequential-level deep model to discriminate infectious corneal disease. The model, constructed using 2284 clinical digital images, achieved a diagnostic accuracy of 80%. Koyama et al.^22^ created a hybrid deep learning-based algorithm utilizing 4306 slit-lamp images for the determination of the probability of causative pathogens in infectious keratitis. Their algorithm demonstrated good performance, with all AUCs exceeding 0.9. In comparison to prior studies, our research had several significant characteristics. First, this study developed a customized AI system (DeepIK) that could mimic the diagnostic process of a human expert in discriminating among bacterial, fungal, viral, amebic, and noninfectious keratitis. Second, this study evaluated DeepIK using 14,245 qualified slit-lamp images from 12 independent clinical centers nationwide and confirmed that DeepIK had good performance and broad generalizability. Third, this study demonstrated that DeepIK could aid junior ophthalmologists in improving their diagnostic performance in discerning the cause of IK, which may allow them to provide prompt and effective treatment strategies in IK patients.

This study has several limitations. First, the development and external test datasets were annotated retrospectively, which may have introduced a certain level of selection bias but the prospective test indicated that this limitation may not be prominent. In addition, owing to the extremely limited number of multiple infection cases, coupled with insufficient diagnostic certainty in the majority of these cases, polymicrobial cases were excluded from the study. Consequently, DeepIK cannot be utilized for the identification of polymicrobial cases. Third, in the AI comparison study, ophthalmologists were provided with a single image, which potentially contained a lesser amount of information compared to the comprehensive data achievable through an in-person slit-lamp examination and a thorough medical history review. Nevertheless, this aligns with the level of information that DeepIK has access to, permitting a direct comparison between these two approaches. In the future, further investigation could explore incorporating information from clinical history, various examination components, and imaging data into a multivariate AI model to enhance diagnostic accuracy.

In summary, our study successfully developed an AI system (DeepIK) exhibiting robust performance in the diagnosis and discrimination of bacterial, fungal, viral, amebic, and noninfectious keratitis from slit-lamp images. This system has the potential to aid ophthalmologists in accurately and promptly identifying the cause of IK, thereby improving patients’ visual prognosis through the initiation of targeted treatment at an early stage.

## Methods

### Development dataset and external test datasets

**Fig. 5:**
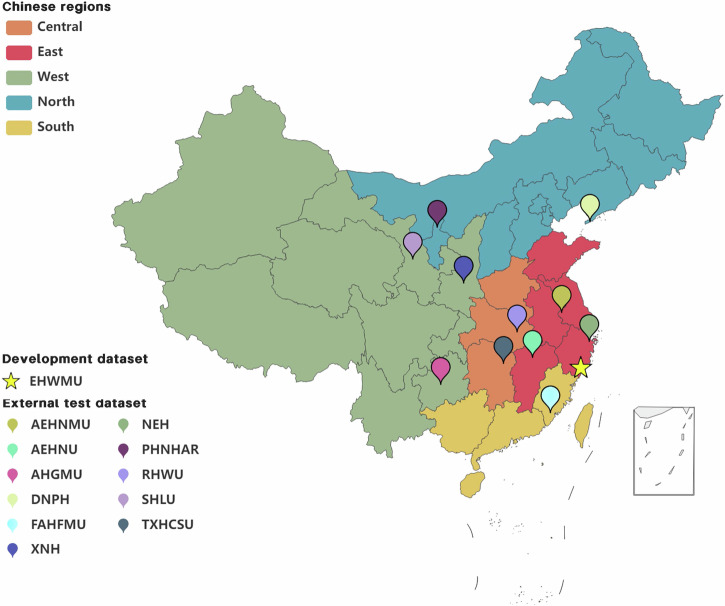
Geographical distribution of the datasets utilized in the model development and evaluation. The images used for the model development were collected from the Eye Hospital of Wenzhou Medical University (EHWMU) and the images used for the model evaluation were collected from 11 other independent clinical centers nationwide. NEH, Ningbo Eye Hospital. DNPH, Dalian No. 3 People’s Hospital. FAHFMU, First Affiliated Hospital of Fujian Medical University. SHLU, Second Hospital of Lanzhou University. AEHNU, Affiliated Eye Hospital of Nanchang University. AEHNMU, Affiliated Eye Hospital of Nanjing Medical University. PHNHAR People’s Hospital of Ningxia Hui Autonomous Region, RHWU Renmin Hospital of Wuhan University, XNH Xi’an No.1 Hospital, TXHCSU Third Xiangya Hospital of Central South University, AHGMU Affiliated Hospital of Guizhou Medical University.

In total, 10,592 slit-lamp images (JPG format) acquired from 6300 patients at EHWMU between May 2007 and October 2021 were utilized to develop a deep learning system. External test datasets including 7206 slit-lamp images (JPG, TIF, PNG, and BMP format) obtained from 11 other clinical centers nationwide (Fig. 5) were employed to further evaluate the efficacy of the system. Both development and external datasets encompassed patients who underwent slit-lamp examination and were eventually diagnosed with various types of keratitis. All images were acquired utilizing digital slit-lamp cameras. Details regarding the specific digital slit-lamp cameras employed at each clinical center are provided in Supplementary Table 19. All the operators of the cameras had received standard training. Multiple images obtained from the same patient, using the diffuse illumination technique with or without slit beam enhancement, were employed in the development of the system for the following reasons: (1) the intensity of light exposure and the positioning of the patient’s eye relative to the slit lamp may vary across different shots.; (2) different images could encompass distinct aspects of the lesion; and (3) the utilization of multiple images could simulate realistic data augmentation, thereby enriching the information available to the algorithm.

### Reference standard and image classification

In this study, poor-quality slit-lamp images were initially filtered out using an AI-based image quality monitoring system which was developed in our previous study^23^. Three ophthalmologists, each having 3 to 6 years of clinical experience, were enlisted to annotate the remaining good-quality images. The determination of the reference standard for each image was made by referencing a definite diagnosis recorded in the hospital information system. This diagnosis was made through a comprehensive consideration of the following pieces of evidence: the medical history, clinical manifestations, corneal assessment (e.g., fluorescein staining and corneal confocal microscopy), laboratory techniques (e.g., corneal scraping smear examination, corneal culture, and PCR), response to definitive therapy (therapeutic diagnosis), and subsequent follow-up visits. The ophthalmologists reviewed all the medical records of each patient to reconfirm the diagnosis through one of the following criteria: (1) detection of a specific pathogen by laboratory techniques (e.g., positive corneal culture) and complete response following definitive therapy; and (2) presence of typical clinical characteristics and complete response following definitive therapy.

This study didn’t use laboratory confirmation of the pathogen as the sole criterion for labeling images of infectious keratitis due to the following reasons:

1. According to the “Herpes Simplex Virus (HSV) Keratitis: A Treatment Guideline” issued by the American Academy of Ophthalmology, in cases of typical HSV epithelial keratitis (dendritic), clinical diagnosis using slit-lamp biomicroscopy examination is reliable, and laboratory tests are generally unnecessary^24^. Laboratory testing is also ineffective in cases of HSV stromal keratitis, as the virus usually cannot be cultured^24^.
2. In contrast to the typically sterile environment of samples obtained from blood or spinal fluid, corneal samples collected from the ocular surface are exposed to a non-sterile environment. Consequently, tests may detect both bacteria that constitute the normal ocular surface microbiome and pathogens responsible for infections^25^. The diagnostic dilemma of infectious keratitis may persist, even with a positive microbiological culture result, due to the lack of a reliable method to differentiate between a pathogen and a commensal organism^25^. This could potentially result in a situation where bacteria identified through culture or PCR may not actually be the causative agent of the disease.
3. Lastly, it is important to note that negative laboratory test results do not necessarily rule out infectious keratitis, as these tests generally have low sensitivity. Developing a deep learning model only using images with positive laboratory test results may introduce selection bias, which results in the model potentially being unable to effectively identify cases of infectious keratitis that have negative laboratory test results. However, the main goal of our study is to develop an approach that has high sensitivity and could identify both laboratory-positive and laboratory-negative infectious keratitis. For this reason, this study also included infectious keratitis cases without positive laboratory test results but with the presence of typical clinical characteristics and complete responses following definitive therapy. Therapeutic diagnosis (typical clinical characteristics with complete response following definitive therapy) has been used by many studies and could act as an accurate alternative approach for determining the labels of infectious keratitis cases with negative laboratory test results^11,15,26^.

Based on the reference standard, the study steering committee categorized the images into five groups: bacterial, fungal, viral, amebic, and noninfectious keratitis. Noninfectious keratitis encompassed cases of keratitis caused by injuries (e.g., fingernail scratches, makeup brushes, and chemical burns), neurotrophic keratitis, immune-mediated keratitis, filamentary keratitis, photokeratitis, etc. The study included only those images that displayed keratitis at the active phase and exhibited sufficient diagnostic certainty.

### Image preprocessing

Prior to the development of the deep learning system, image standardization was conducted. All slit-lamp images underwent pixel value normalization to achieve a range of 0–1, and their sizes were resized to a resolution of 224 × 224 pixels. In addition, to enhance the diversity of the training dataset, prevent overfitting and bias, and improve the generalization of the model, several image augmentation techniques were employed. These techniques were as follows: (1) random crop, (2) random contrast, (3) random brightness, (4) random rotations around the image center, and (5) random horizontal and vertical flips (Supplementary Table 20). The training dataset was ultimately expanded to six times its original size, growing from 7362 to 44,172 instances.

### Development of a deep learning system

The development dataset was randomly divided into training, validation, and internal test datasets, with proportions of 70%, 15%, and 15% respectively. All images associated with the same patient should fall into one split to prevent data leakage and biased evaluation of performance. Three CNN architectures, DenseNet121, InceptionResNetV2, and Swin-Transformer, were employed for model training. To establish initial weights and leverage existing knowledge, the model underwent pre-training on the ImageNet Large Scale Visual Recognition Challenge, a comprehensive database containing 1.28 million images categorized across 1000 object categories^27^.

To make AI mimic the diagnostic process of a human expert in identifying the cause of keratitis (Fig. 4a), applicable customization of the algorithm was conducted. Specifically, the algorithm DenseNet121 underwent a modification where the final classification layer was exchanged with a dual-task layer housing two classifiers: the first classifier was used to distinguish between infectious and noninfectious keratitis; and the second classifier leveraged the prior knowledge learned from the first classifier to further classify keratitis into bacterial, fungal, viral, amebic, and noninfectious keratitis (Fig. 4b). As the outcome of the first classifier can be utilized as the prior for the second classifier, this strategy, which is similar to that used by a human ophthalmologist, may improve the final performance of the five-category classification model. This customized algorithm is named DeepIK in our study. DeepIK was trained by minimizing the loss function (cross-entropy loss) consisting of two types of losses, which can be displayed as follows: *Loss* = α*classifier_1_loss + (1-α) * classifier_2_loss. Here, *Loss* denotes the overall loss of the selected model, while *classifier_1_loss* and *classifier_2_loss* indicate the losses corresponding to classifier 1 and classifier 2, respectively. *α* is a weight parameter that is employed to balance the two losses.

PyTorch was utilized as the backend framework for training all the algorithms, which were executed on four Nvidia 2080TI graphics processing units (GPUs). To train the models, a mini-batch size of 32 was assigned for each GPU, resulting in 128 images processed in each iteration. The trainable parameters were updated based on the mean value computed from these samples. The training process made use of the adaptive moment estimation (ADAM) optimizer, with weight decay, β1, and β2 set to 1e-4, 0.9, and 0.999, respectively. The initial learning rate was set to 0.001 and was subsequently decreased by a factor of one-tenth every 20 epochs. This study employed the cost-sensitive method and assigned a larger weight (3 times the weight of viral/noninfectious keratitis) to the class of amebic keratitis which had a smaller sample size. As distinguishing bacterial keratitis from fungal keratitis is difficult, this study also assigned larger weights to these two categories (bacterial keratitis: 5 times the weight of viral/noninfectious keratitis, and fungal keratitis: 3 times the weight of viral/noninfectious keratitis). The specific steps of this approach have been elaborated in our previous study^28^. The corresponding implementation details can be found in the source code (https://github.com/jiangjiewei/DeepIK). Each model underwent training for 80 epochs, with the loss and accuracy being assessed on a validation dataset at each epoch to monitor the model’s performance. The training loss curves for each model are shown in Supplementary Fig. 21. The model exhibiting the highest accuracy on the validation dataset was ultimately applied to an internal test dataset.

The external test datasets were used to further evaluate the efficacy of this five-category classification model. The prospective pilot study was conducted in EHWMU (Wenzhou from November 2021 to October 2022). This prospective dataset is independent of the development dataset that was used to establish DeepIK. The process of developing and evaluating the model is detailed in Supplementary Fig. 22. The information regarding each trained model, such as the trainable parameters, size, and running time, is summarized in Supplementary Table 18. In a two-dimensional space, the t-SNE technique was employed to showcase the embedding features acquired by the model for each category^29^.

### Interpretation of AI diagnosis

To facilitate comprehension of the operational principles of the deep learning system among clinicians, visual explanations were generated using Gradient-weighted Class Activation Mapping (Grad-CAM) for images from test datasets. This technique employed the gradients of a target concept, which propagate into the final convolutional layer, to generate a localization map that emphasizes important regions in the image for concept prediction^30^. Based on this method, a heatmap was produced to elucidate the reasoning of the system in diagnosing bacterial, fungal, viral, amebic, and noninfectious keratitis. Redder regions in heatmaps indicated more important features in the system’s decision.

### Analysis of misclassification

In a post-hoc analysis, this study counted the number of images that DeepIK misclassified for each type of keratitis and investigated the type of misclassification. In addition, the relationship between the system’s error rate and its predicted probability was analyzed.

### DeepIK versus ophthalmologists

To compare the performance of DeepIK with that of ophthalmologists, this study created a contest dataset of 250 images. This dataset included 50 images each of bacterial, fungal, viral, amebic, and noninfectious keratitis, which were randomly selected from external test datasets. The images were mixed up and deidentified prior to the ophthalmologists’ assessment. Four practicing ophthalmologists, not engaged in the image annotation task, were recruited to participate in the comparative study. They were categorized into two groups: the junior group, with 6 to 15 years of experience in diagnosing and treating corneal diseases, and the senior group, with 16 to 25 years of experience in the same field. They were requested to independently assign each image to one of the following 5 categories: bacterial, fungal, viral, amebic, and noninfectious keratitis and their outputs were solely based on information derived from images. No other clinical information was provided. To mitigate bias from the competition, the ophthalmologists were not informed that they were in competition with DeepIK. In addition, to avoid ophthalmologists being swayed by variations in the prevalence of different types of keratitis during image classification, all ophthalmologists were notified that, in this dataset, the proportions of various types of keratitis might be similar or inconsistent. A weighted error, determined by penalty scores, was employed to assess the clinical performance of both DeepIK and the ophthalmologists. This study assigned a score of 2 to the misdiagnosis of IK (bacterial, fungal, viral, and amebic keratitis) as noninfectious keratitis, considering it may result in a more severe outcome compared to misdiagnosing noninfectious keratitis as infectious, which was given a score of 1. Besides, scores for misdiagnosing the remaining categories were designated as 1.

An additional study was conducted to examine the influence of DeepIK on enhancing the performance of the two junior ophthalmologists. In the re-examination of the identical slit-lamp image four weeks after the initial assessment, DeepIK supplied the predicted probability of each keratitis type for every image. Subsequently, the junior ophthalmologists were prompted to re-diagnose the cases, enabling an assessment of the influence of our system on their diagnostic performance in comparison to the reference standard.

### Statistical analysis

To report performance characteristics for this 5-class classification system, this study employed the one-vs-rest strategy and computed metrics such as accuracy, sensitivity, specificity, and AUC. The two-sided 95% CIs for accuracy, sensitivity, and specificity were determined using the Wilson Score method through the Statsmodels package (version 0.11.1). For calculating the 95% CI of AUC, the Empirical Bootstrap method with 1000 random replicates was employed. ROC curves were constructed utilizing the Matplotlib (version 3.3.1) and Scikit-learn (version 0.23.2) packages. Confusion matrices were leveraged to illustrate the classification results. The concordance between the outputs of the deep learning system and the reference standard was evaluated using unweighted Cohen kappa scores. This study utilized a McNemar test to compare the accuracies, sensitivities, and specificities between the system and the ophthalmologists. Statistical analysis was performed using Python 3.7.8 (Wilmington, Delaware, USA), with statistical significance set at a two-sided P-value below 0.05. The study was registered with ClinicalTrials.gov (NCT05538793).

### Reporting summary

Further information on research design is available in the Nature Research Reporting Summary linked to this article.

### Supplementary information

Supplementary Information

Reporting Summary

## Data Availability

The data substantiating the main findings of this study can be found in the manuscript and its Supplementary Information. Due to regulations imposed by hospitals and concerns regarding patient privacy, the raw datasets from individual clinical centers cannot be provided. Anonymized data is accessible for research purposes and can be obtained from the corresponding authors upon a reasonable request.

## Code availability

The custom codes for DeepIK development and evaluation in this study are accessible on GitHub (https://github.com/jiangjiewei/DeepIK).
