# Supplementary material for: Deep learning for multi-type infectious keratitis diagnosis: A nationwide, cross-sectional, multicenter study
Source: NPJ Digit Med. 2024 Jul 6;7:181. doi: 10.1038/s41746-024-01174-w (PMC11227533; doi:10.1038/s41746-024-01174-w)
Supplement: Supplementary file 1 — Supplementary Information [file 41746_2024_1174_MOESM1_ESM.pdf]

Supplementary Information

Deep learning for multi-type infectious keratitis diagnosis: a nationwide, cross-sectional, multicenter study

Contents:

**Supplementary item**	Page
**Supplementary Figures**	3
Supplementary Figure 1	3
Supplementary Figure 2	4
Supplementary Figure 3	5
Supplementary Figure 4	6
Supplementary Figure 5	8
Supplementary Figure 6	10
Supplementary Figure 7	12
Supplementary Figure 8	14
Supplementary Figure 9	16
Supplementary Figure 10	18
Supplementary Figure 11	20
Supplementary Figure 12	22
Supplementary Figure 13	24
Supplementary Figure 14	26
Supplementary Figure 15	28
Supplementary Figure 16	29
Supplementary Figure 17	30
Supplementary Figure 18	31
Supplementary Figure 19	32
Supplementary Figure 20	33
Supplementary Figure 21	34
Supplementary Figure 22	35
**Supplementary Tables**	36
Supplementary Table 1	36
Supplementary Table 2	37
Supplementary Table 3	38
Supplementary Table 4	39
Supplementary Table 5	40
Supplementary Table 6	41
Supplementary Table 7	42
Supplementary Table 8	43
Supplementary Table 9	44
Supplementary Table 10	45
Supplementary Table 11	46
Supplementary Table 12	47

Supplementary Table 13	48
Supplementary Table 14	49
Supplementary Table 15	50
Supplementary Table 16	51
Supplementary Table 17	53
Supplementary Table 18	54
Supplementary Table 19	55
Supplementary Table 20	56

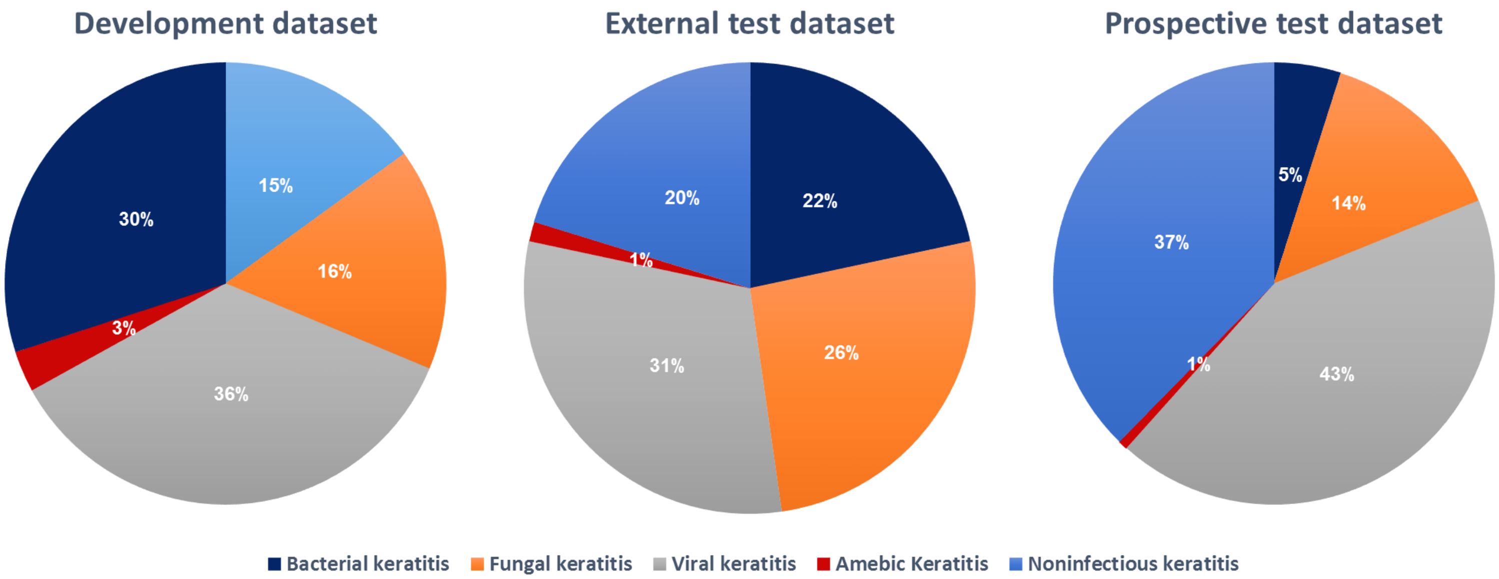

Supplementary Figure 1. Proportions of each type of keratitis in the development dataset and the external and prospective test datasets. In the development dataset, external test dataset, and prospective test dataset, the proportions of bacterial keratitis were 30%, 22%, and 5%, respectively; the proportions of fungal keratitis were 16%, 26%, and 14%, respectively; the proportions of viral keratitis were 36%, 31%, and 43%, respectively; the proportions of amebic keratitis were 3%, 1%, and 1%, respectively; and the proportions of noninfectious keratitis were 15%, 20%, and 37%, respectively.

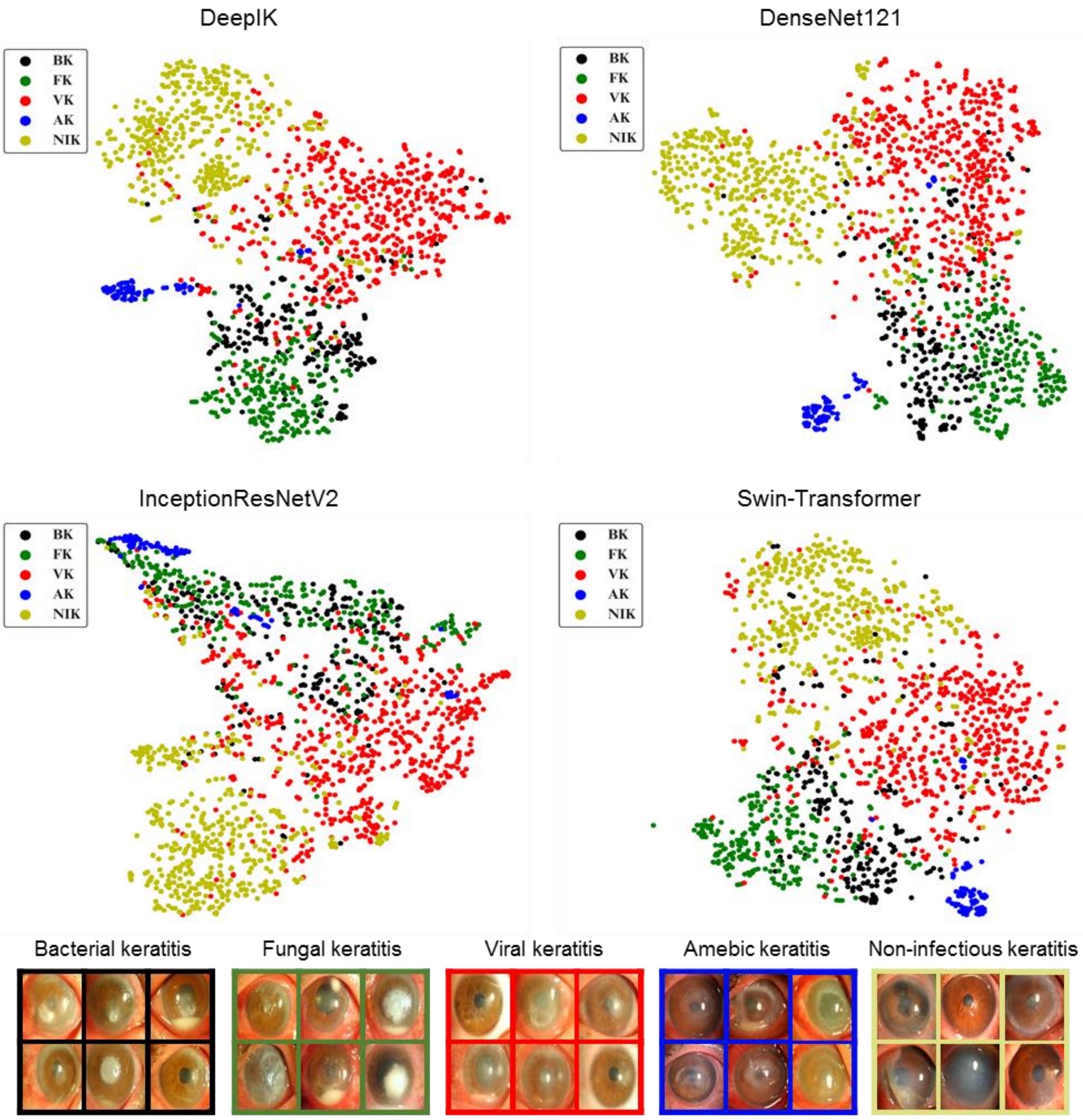

Supplementary Figure 2. Visualization of embedding features learned by deep learning algorithms with t-SNE from the internal test dataset. Embedding features are represented as dots to show the class distribution. Different colored dot clouds indicate different classes. The classes are bacterial keratitis (black), fungal keratitis (green), viral keratitis (red), amebic keratitis (blue), and noninfectious keratitis (yellow).

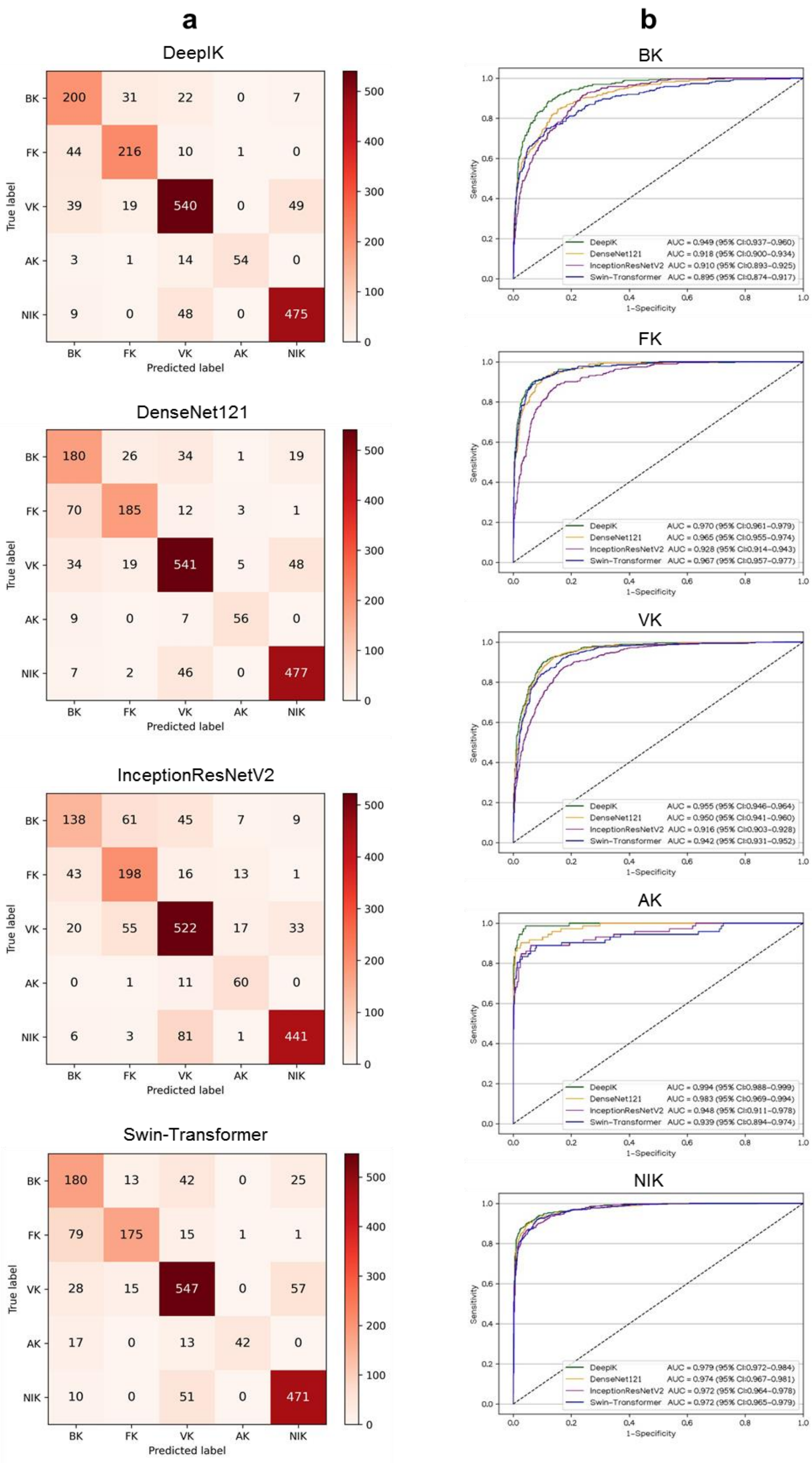

Supplementary Figure 3. Performance of deep learning algorithms for the diagnoses of bacterial, fungal, viral, amebic, and noninfectious keratitis in an internal test dataset. a Confusion matrices showing the accuracies of four deep learning algorithms. **b** Receiver operating characteristic curves of four deep learning algorithms for the classification of bacterial, fungal, viral, amebic, and noninfectious keratitis. BK, bacterial keratitis. FK, fungal keratitis. VK, viral keratitis. AK, amebic keratitis. NIK, noninfectious keratitis.

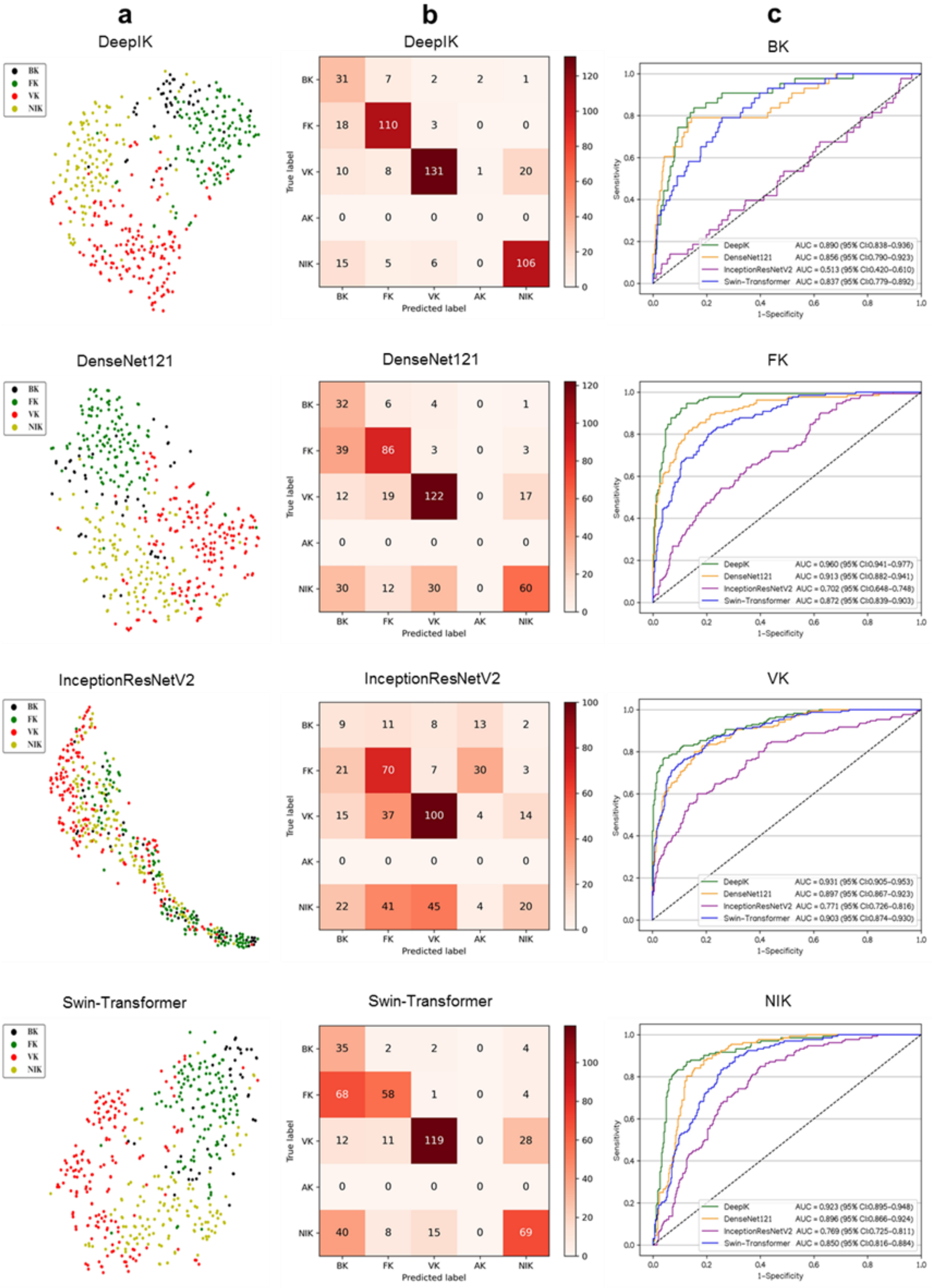

Supplementary Figure 4. Performance of deep learning algorithms for the diagnosis of bacterial, fungal, viral, amebic, and noninfectious keratitis in the NEH external test dataset. **a** Embedding features learned by the deep learning algorithms are projected in two dimensions with t-SNE. The embedding features are represented as dots to show the class distribution. Different colored dot clouds indicate different classes. **b** Confusion matrices showing the accuracies of four deep learning algorithms. **c** Receiver operating characteristic curves of four deep learning algorithms for the classification of bacterial, fungal, viral, amebic, and noninfectious keratitis. NEH,

Ningbo Eye Hospital. BK, bacterial keratitis. FK, fungal keratitis. VK, viral keratitis. AK, amebic keratitis. NIK, noninfectious keratitis.

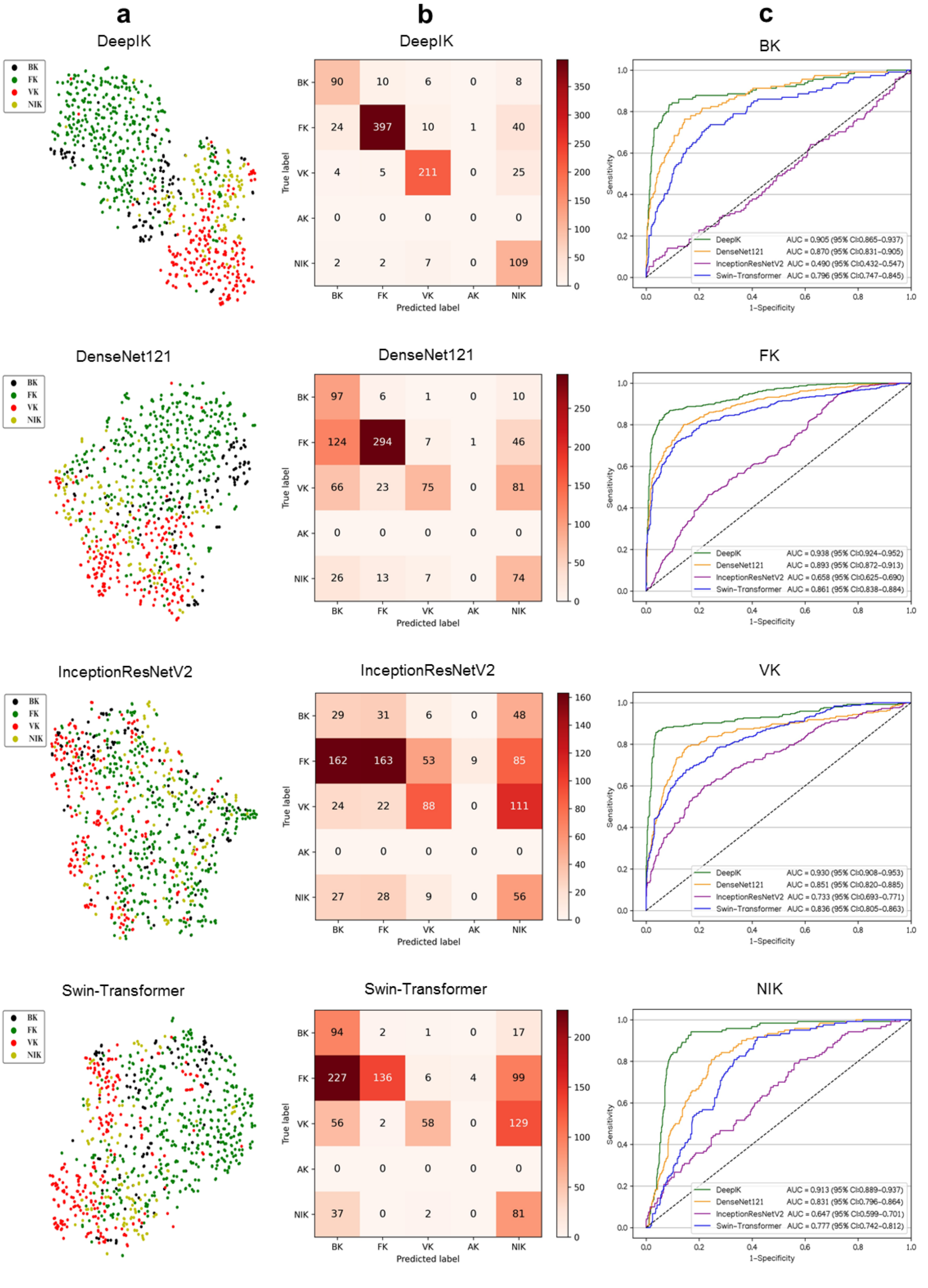

Supplementary Figure 5. Performance of deep learning algorithms for the diagnosis of bacterial, fungal, viral, amebic, and noninfectious keratitis in the DNPH external test dataset. **a** Embedding features learned by the deep learning algorithms are projected in two dimensions with t-SNE. The embedding features are represented as dots to show the class distribution. Different colored dot clouds indicate different classes. **b** Confusion matrices showing the accuracies of four deep learning algorithms. **c** Receiver operating characteristic curves of four deep learning

algorithms for the classification of bacterial, fungal, viral, amebic, and noninfectious keratitis. DNPH, Dalian No.3 People's Hospital. BK, bacterial keratitis. FK, fungal keratitis. VK, viral keratitis. AK, amebic keratitis. NIK, noninfectious keratitis.

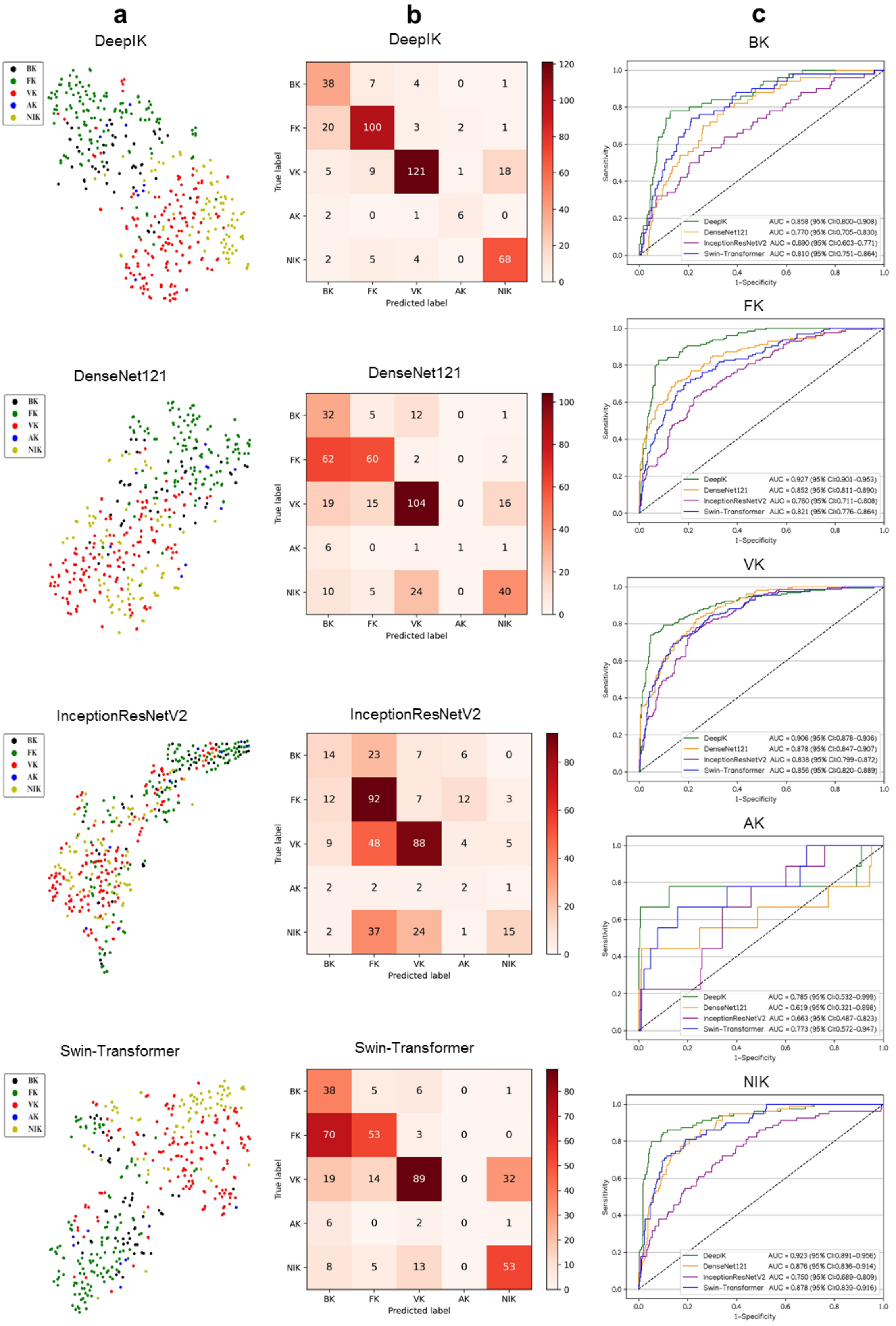

Supplementary Figure 6. Performance of deep learning algorithms for the diagnosis of bacterial, fungal, viral, amebic, and noninfectious keratitis in the FAHFMU external test dataset. a Embedding features learned by the deep learning algorithms are projected in two dimensions with t-SNE. The embedding features are represented as dots to show the class

distribution. Different colored dot clouds indicate different classes. **b** Confusion matrices showing the accuracies of four deep learning algorithms. **c** Receiver operating characteristic curves of four deep learning algorithms for the classification of bacterial, fungal, viral, amebic, and noninfectious keratitis. FAHFMU, First Affiliated Hospital of Fujian Medical University. BK, bacterial keratitis. FK, fungal keratitis. VK, viral keratitis. AK, amebic keratitis. NIK, noninfectious keratitis.

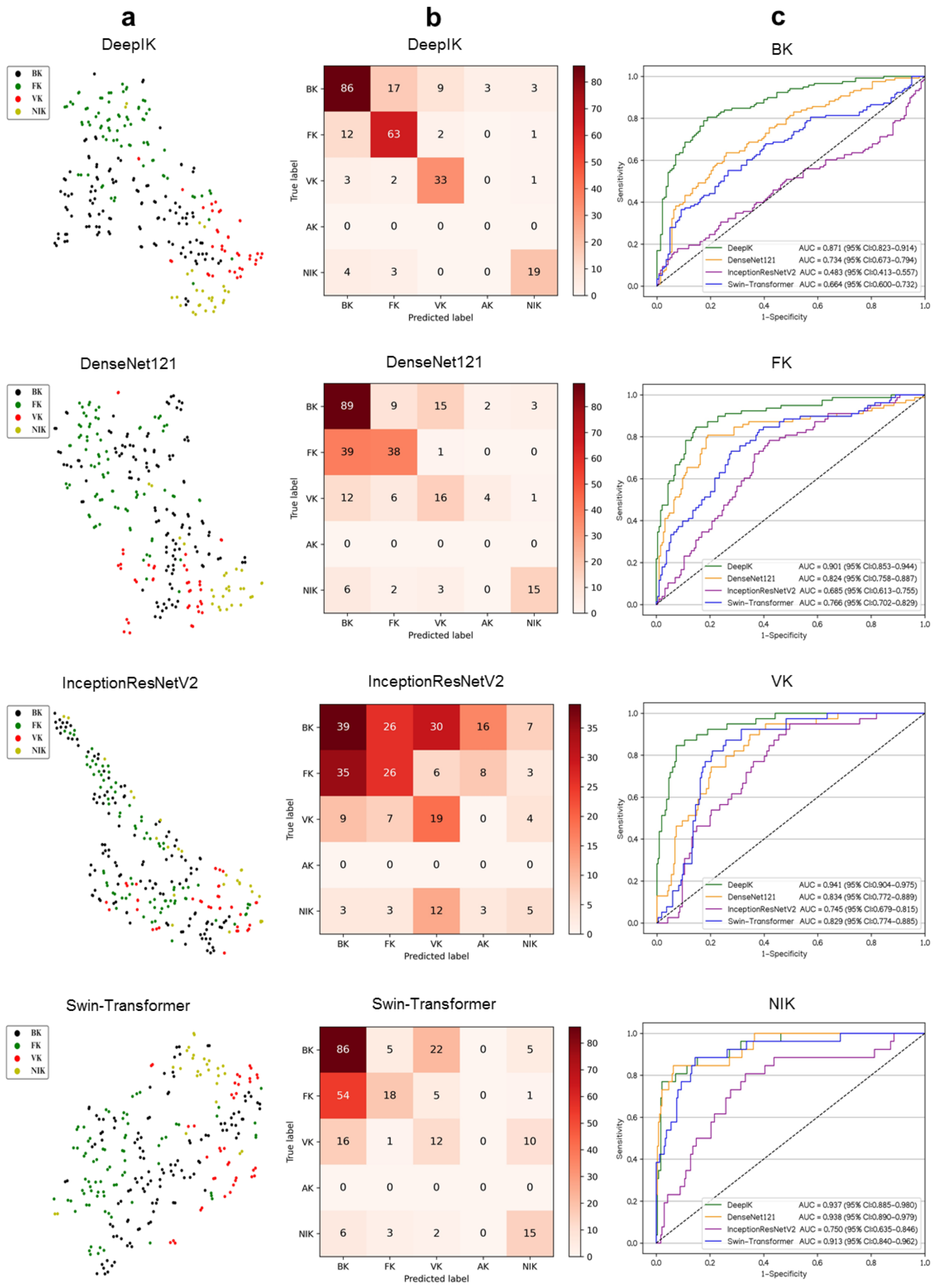

Supplementary Figure 7. Performance of deep learning algorithms for the diagnosis of bacterial, fungal, viral, amebic, and noninfectious keratitis in the SHLU external test dataset. **a** Embedding features learned by the deep learning algorithms are projected in two dimensions with t-SNE. The embedding features are represented as dots to show the class distribution. Different colored dot clouds indicate different classes. **b** Confusion matrices showing the accuracies of four deep learning algorithms. **c** Receiver operating characteristic curves of four deep learning

algorithms for the classification of bacterial, fungal, viral, amebic, and noninfectious keratitis. SHLU, Second Hospital of Lanzhou University. BK, bacterial keratitis. FK, fungal keratitis. VK, viral keratitis. AK, amebic keratitis. NIK, noninfectious keratitis.

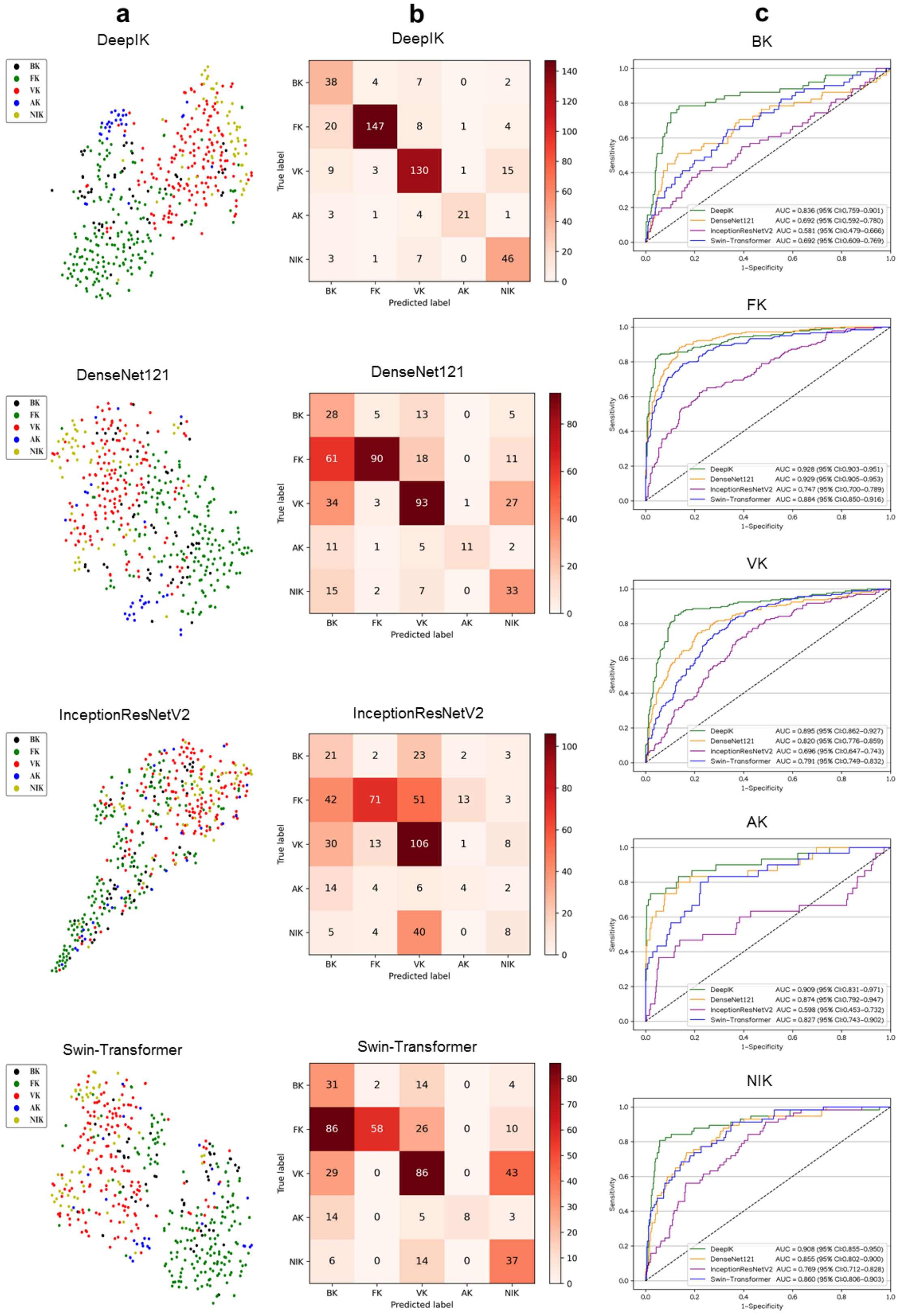

Supplementary Figure 8. Performance of deep learning algorithms for the diagnosis of bacterial, fungal, viral, amebic, and noninfectious keratitis in the AEHNU external test dataset. **a** Embedding features learned by the deep learning algorithms are projected in two dimensions with t-SNE. The embedding features are represented as dots to show the class distribution. Different

colored dot clouds indicate different classes. **b** Confusion matrices showing the accuracies of four deep learning algorithms. **c** Receiver operating characteristic curves of four deep learning algorithms for the classification of bacterial, fungal, viral, amebic, and noninfectious keratitis. AEHNU, Affiliated Eye Hospital of Nanchang University. BK, bacterial keratitis. FK, fungal keratitis. VK, viral keratitis. AK, amebic keratitis. NIK, noninfectious keratitis.

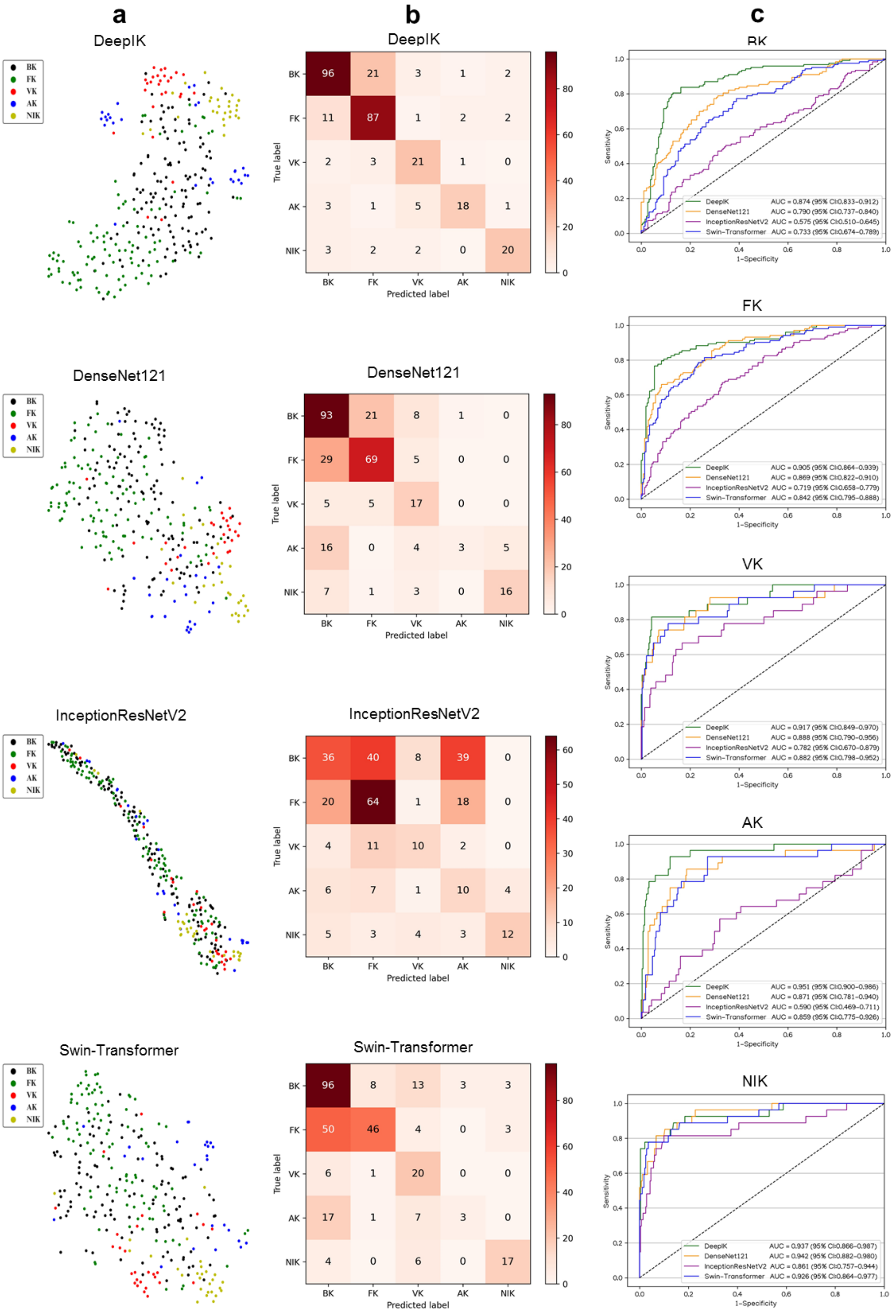

Supplementary Figure 9. Performance of deep learning algorithms for the diagnosis of bacterial, fungal, viral, amebic, and noninfectious keratitis in the AEHNMU external test dataset. a Embedding features learned by the deep learning algorithms are projected in two dimensions with t-SNE. The embedding features are represented as dots to show the class

distribution. Different colored dot clouds indicate different classes. **b** Confusion matrices showing the accuracies of four deep learning algorithms. **c** Receiver operating characteristic curves of four deep learning algorithms for the classification of bacterial, fungal, viral, amebic, and noninfectious keratitis. AEHNMU, Affiliated Eye Hospital of Nanjing Medical University. BK, bacterial keratitis. FK, fungal keratitis. VK, viral keratitis. AK, amebic keratitis. NIK, noninfectious keratitis.

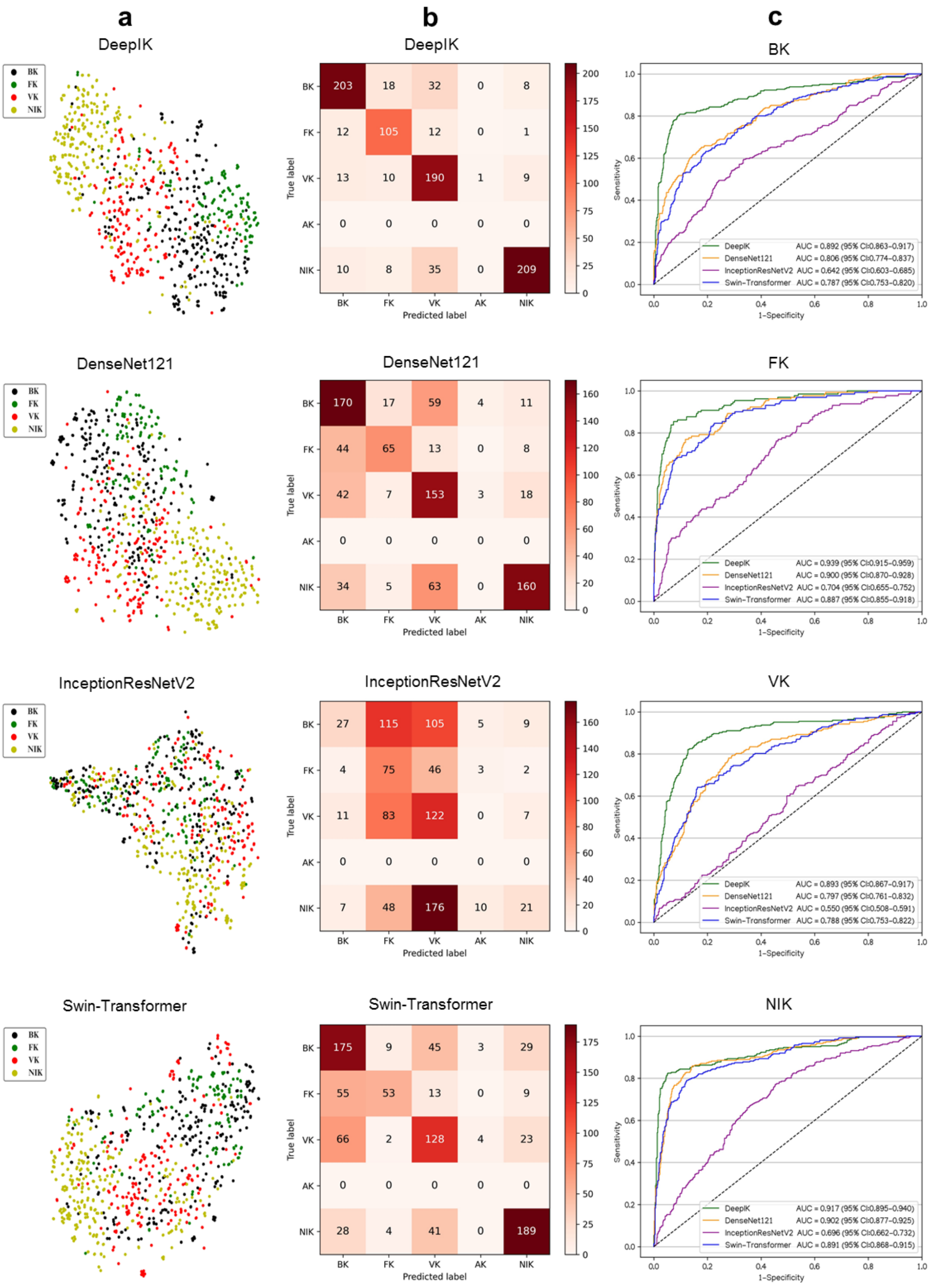

Supplementary Figure 10. Performance of deep learning algorithms for the diagnosis of bacterial, fungal, viral, amebic, and noninfectious keratitis in the PHNHAR external test dataset. **a** Embedding features learned by the deep learning algorithms are projected in two dimensions with t-SNE. The embedding features are represented as dots to show the class distribution. Different colored dot clouds indicate different classes. **b** Confusion matrices showing the accuracies of four deep learning algorithms. **c** Receiver operating characteristic curves of four

deep learning algorithms for the classification of bacterial, fungal, viral, amebic, and noninfectious keratitis. PHNHAR, People’s Hospital of Ningxia Hui Autonomous Region. BK, bacterial keratitis. FK, fungal keratitis. VK, viral keratitis. AK, amebic keratitis. NIK, noninfectious keratitis.

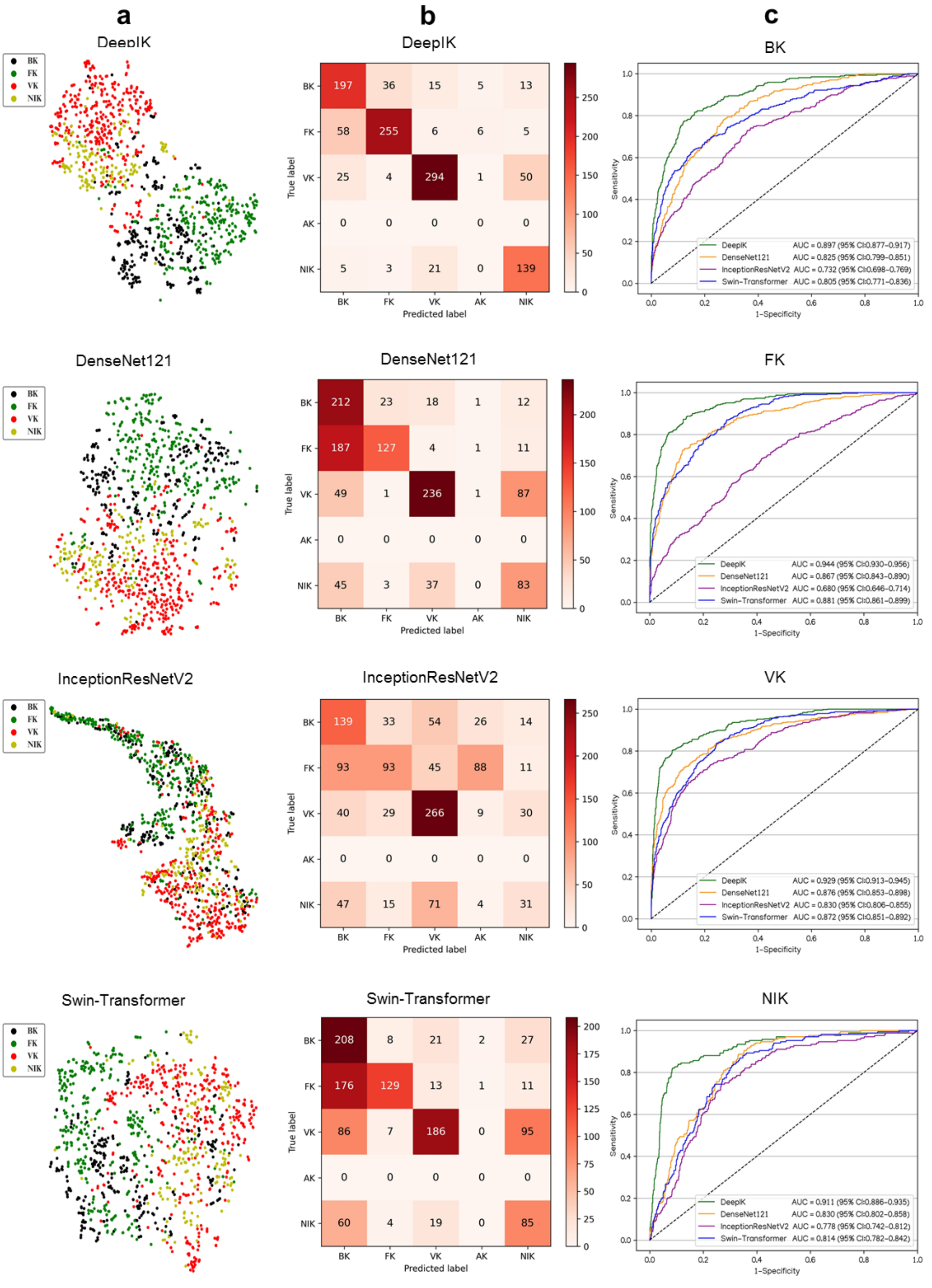

Supplementary Figure 11. Performance of deep learning algorithms for the diagnosis of bacterial, fungal, viral, amebic, and noninfectious keratitis in the RHWU external test dataset. **a** Embedding features learned by the deep learning algorithms are projected in two dimensions with t-SNE. The embedding features are represented as dots to show the class distribution. Different colored dot clouds indicate different classes. **b** Confusion matrices showing the accuracies of four deep learning algorithms. **c** Receiver operating characteristic curves of four deep learning

algorithms for the classification of bacterial, fungal, viral, amebic, and noninfectious keratitis. RHWU, Renmin Hospital of Wuhan University. BK, bacterial keratitis. FK, fungal keratitis. VK, viral keratitis. AK, amebic keratitis. NIK, noninfectious keratitis.

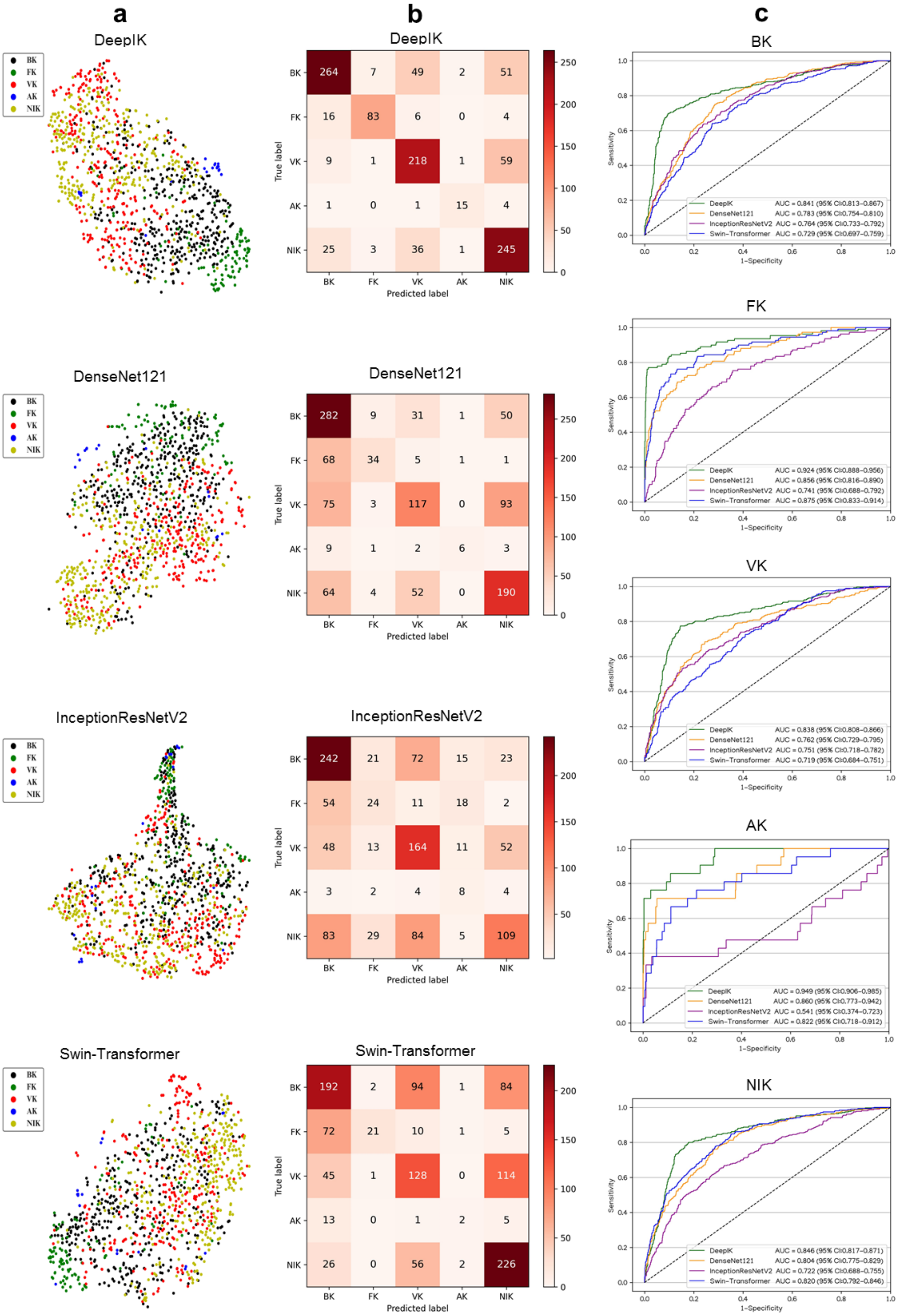

Supplementary Figure 12. Performance of deep learning algorithms for the diagnosis of bacterial, fungal, viral, amebic, and noninfectious keratitis in the XNH external test dataset. **a** Embedding features learned by the deep learning algorithms are projected in two dimensions with t-SNE. The embedding features are represented as dots to show the class distribution. Different

colored dot clouds indicate different classes. **b** Confusion matrices showing the accuracies of four deep learning algorithms. **c** Receiver operating characteristic curves of four deep learning algorithms for the classification of bacterial, fungal, viral, amebic, and noninfectious keratitis. XNH, Xi'an No.1 Hospital. BK, bacterial keratitis. FK, fungal keratitis. VK, viral keratitis. AK, amebic keratitis. NIK, noninfectious keratitis.

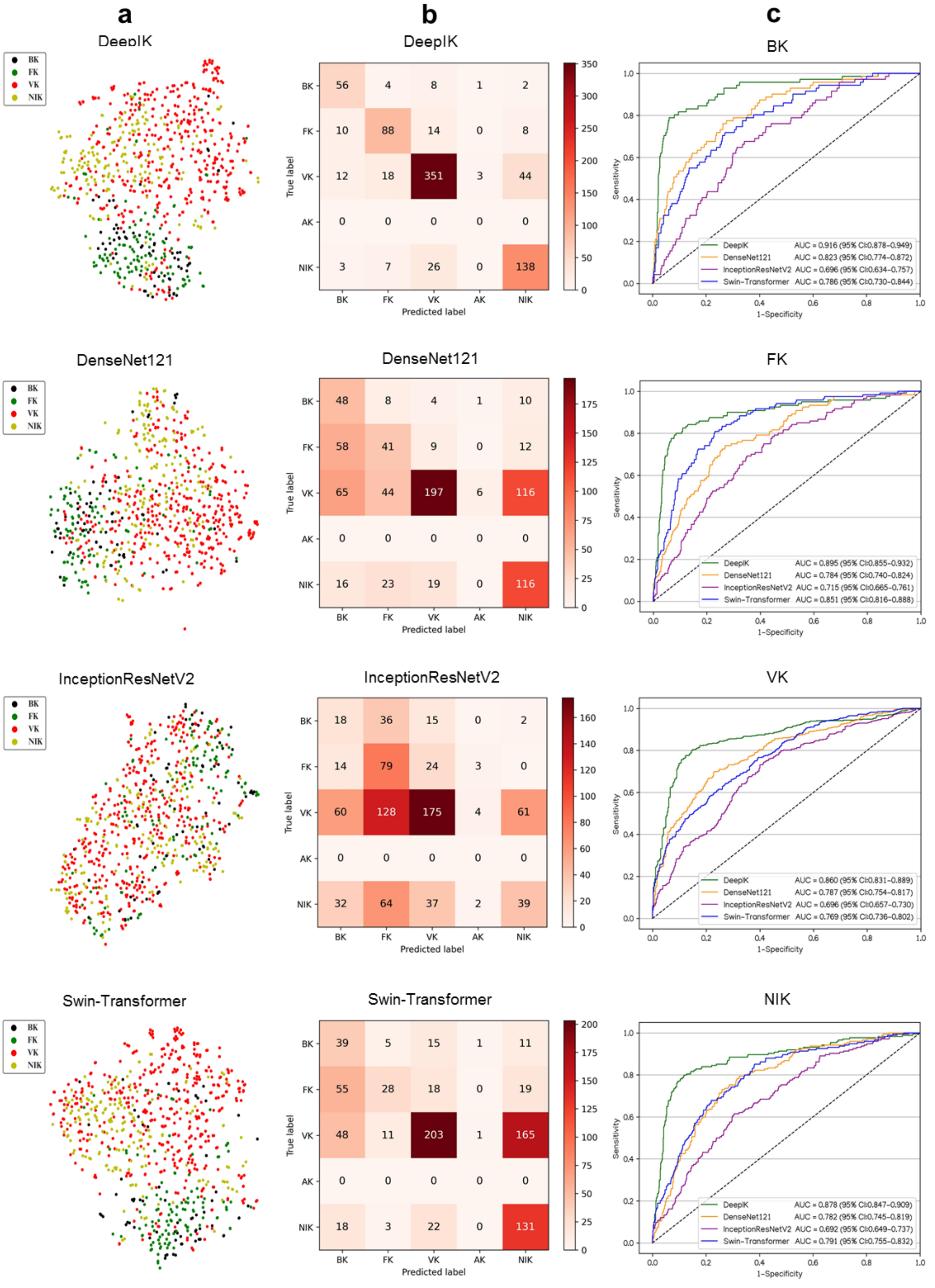

Supplementary Figure 13. Performance of deep learning algorithms for the diagnosis of bacterial, fungal, viral, amebic, and noninfectious keratitis in the TXHCSU external test dataset. a Embedding features learned by the deep learning algorithms are projected in two dimensions with t-SNE. The embedding features are represented as dots to show the class distribution. Different colored dot clouds indicate different classes. **b** Confusion matrices showing the accuracies of four deep learning algorithms. **c** Receiver operating characteristic curves of four

deep learning algorithms for the classification of bacterial, fungal, viral, amebic, and noninfectious keratitis. TXHCSU, Third Xiangya Hospital of Central South University. BK, bacterial keratitis. FK, fungal keratitis. VK, viral keratitis. AK, amebic keratitis. NIK, noninfectious keratitis.

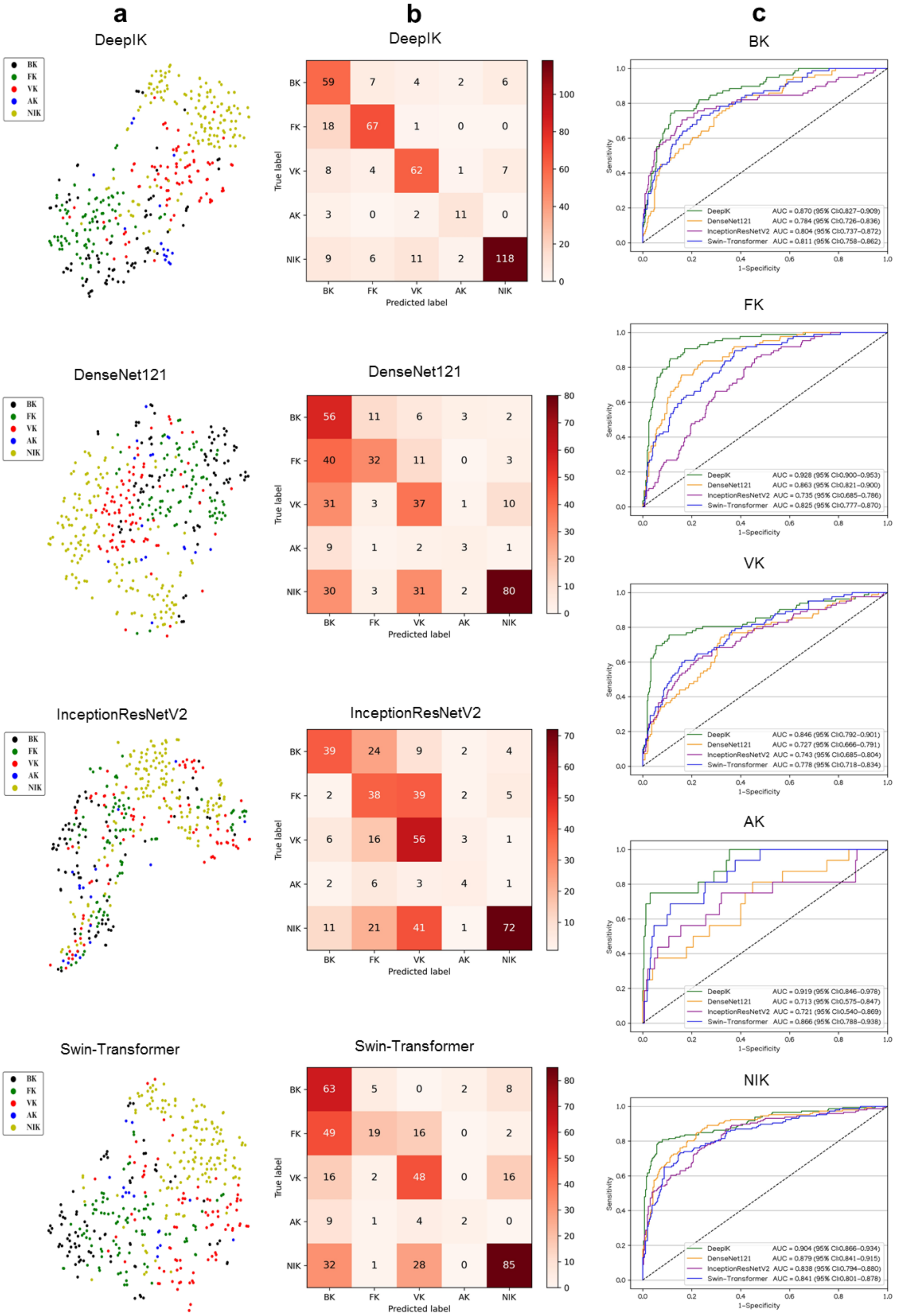

Supplementary Figure 14. Performance of deep learning algorithms for the diagnosis of bacterial, fungal, viral, amebic, and noninfectious keratitis in the AHGMU external test dataset. a Embedding features learned by the deep learning algorithms are projected in two dimensions with t-SNE. The embedding features are represented as dots to show the class

distribution. Different colored dot clouds indicate different classes. **b** Confusion matrices showing the accuracies of four deep learning algorithms. **c** Receiver operating characteristic curves of four deep learning algorithms for the classification of bacterial, fungal, viral, amebic, and noninfectious keratitis. AHGMU, Affiliated Hospital of Guizhou Medical University. BK, bacterial keratitis. FK, fungal keratitis. VK, viral keratitis. AK, amebic keratitis. NIK, noninfectious keratitis.

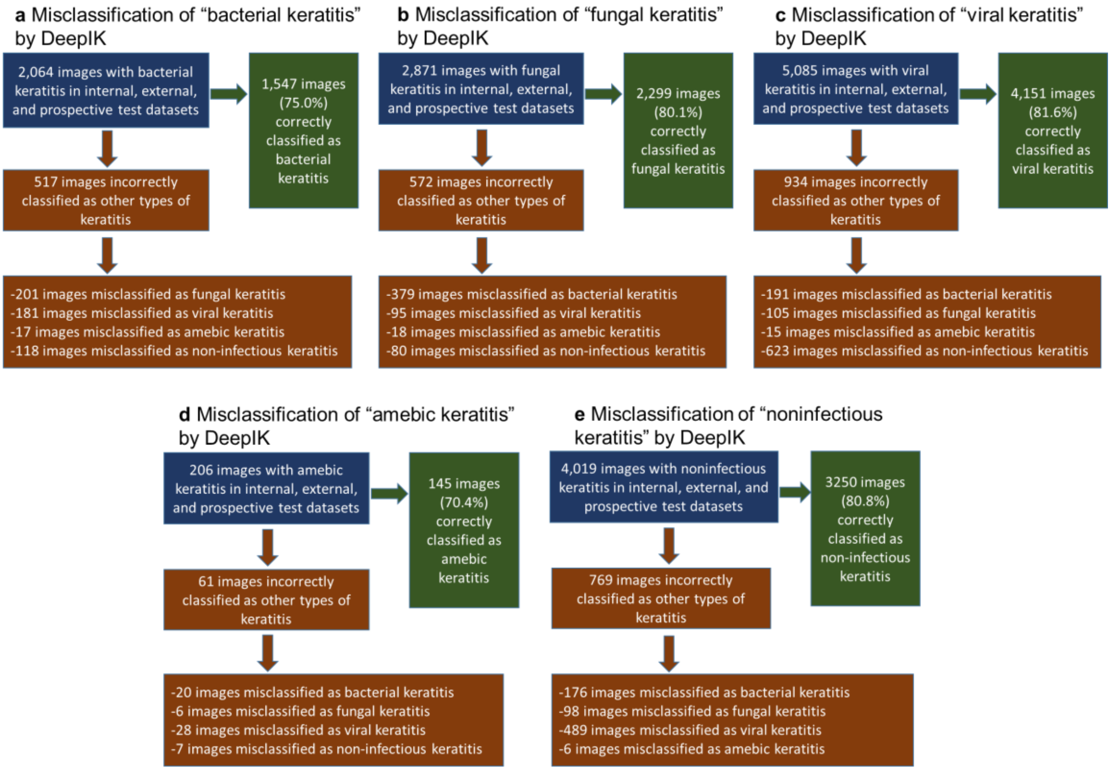

Supplementary Figure 15. Errors in classification by DeepIK in internal, external, and prospective test datasets. a Misclassification of "bacterial keratitis" by DeepIK. **b** Misclassification of "fungal keratitis" by DeepIK. **c** Misclassification of "viral keratitis" by DeepIK. **d** Misclassification of "amebic keratitis" by DeepIK. **e** Misclassification of "noninfectious keratitis" by DeepIK.

a BK incorrectly classified as other types of keratitis<br>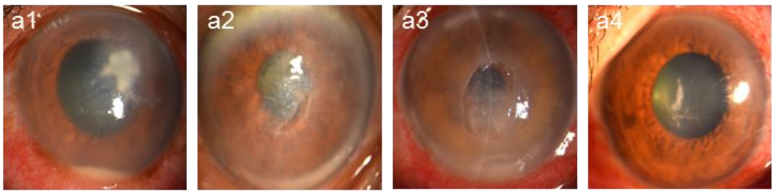	a1, BK misclassified as FK.<br>a2, BK misclassified as VK.<br>a3, BK misclassified as AK.<br>a4, BK misclassified as NIK
b FK incorrectly classified as other types of keratitis<br>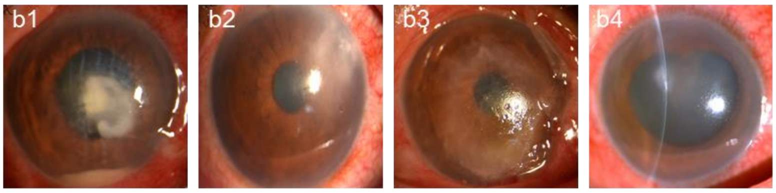	b1, FK misclassified as BK.<br>b2, FK misclassified as VK.<br>b3, FK misclassified as AK.<br>b4, FK misclassified as NIK.
c VK incorrectly classified as other types of keratitis<br>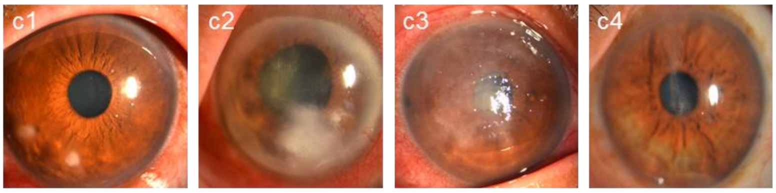	c1, VK misclassified as BK.<br>c2, VK misclassified as FK.<br>c3, VK misclassified as AK.<br>c4, VK misclassified as NIK.
d AK incorrectly classified as other types of keratitis<br>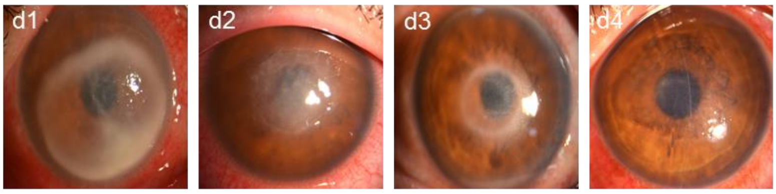	d1, AK misclassified as BK.<br>d2, AK misclassified as FK.<br>d3, AK misclassified as VK.<br>d4, AK misclassified as NIK.
e NIK incorrectly classified as other types of keratitis<br>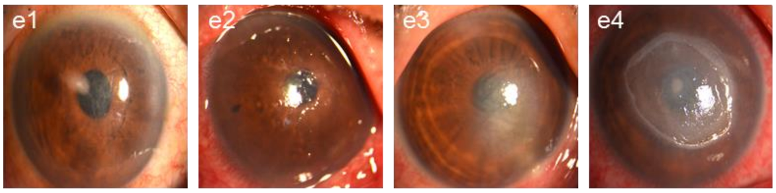	e1, NIK misclassified as BK.<br>e2, NIK misclassified as FK.<br>e3, NIK misclassified as VK.<br>e4, NIK misclassified as AK.

Supplementary Figure 16. Representative examples of misclassified images by DeepIK. a Misclassified images of bacterial keratitis (BK). **b** Misclassified images of fungal keratitis (FK). **c** Misclassified images of viral keratitis (VK). **d** Misclassified images of amebic keratitis (AK). **e** Misclassified images of noninfectious keratitis (NIK).

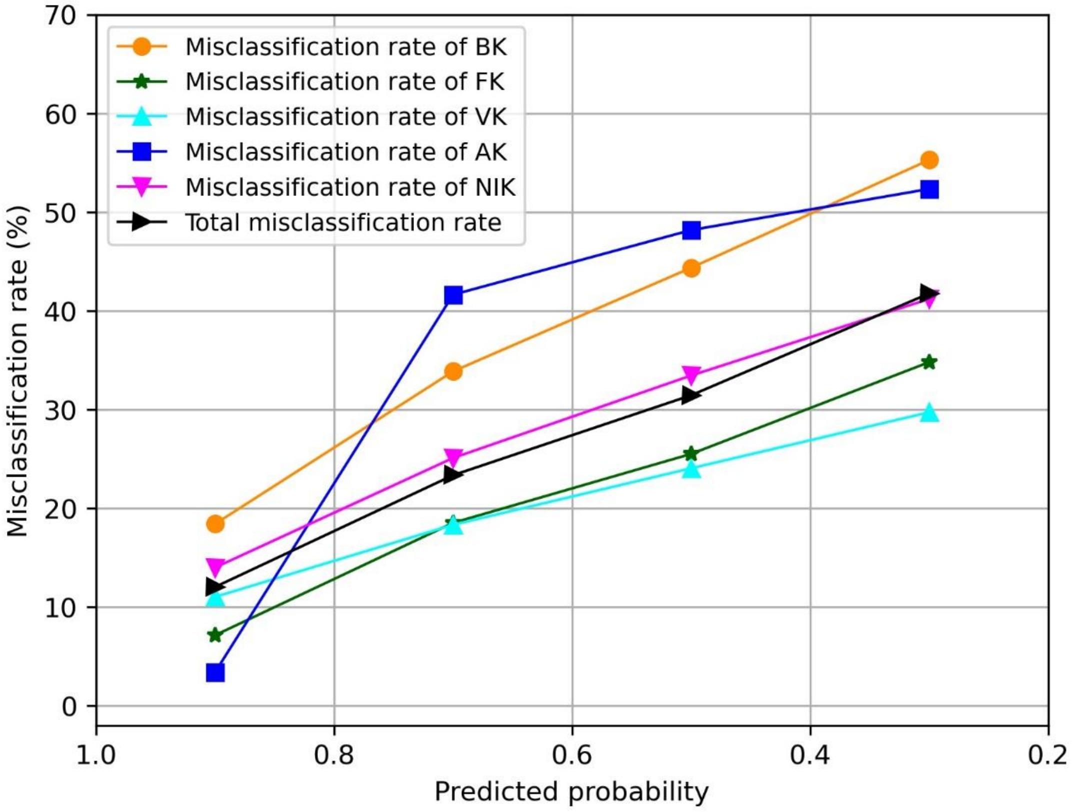

Supplementary Figure 17. Correlation between the predicted probabilities of DeepIK and misclassification rates. The misclassification rate refers to the proportion of misclassified images within each predicted probability interval delimited by the breaking points. BK, bacterial keratitis. FK, fungal keratitis. VK, viral keratitis. AK, amebic keratitis. NIK, noninfectious keratitis.

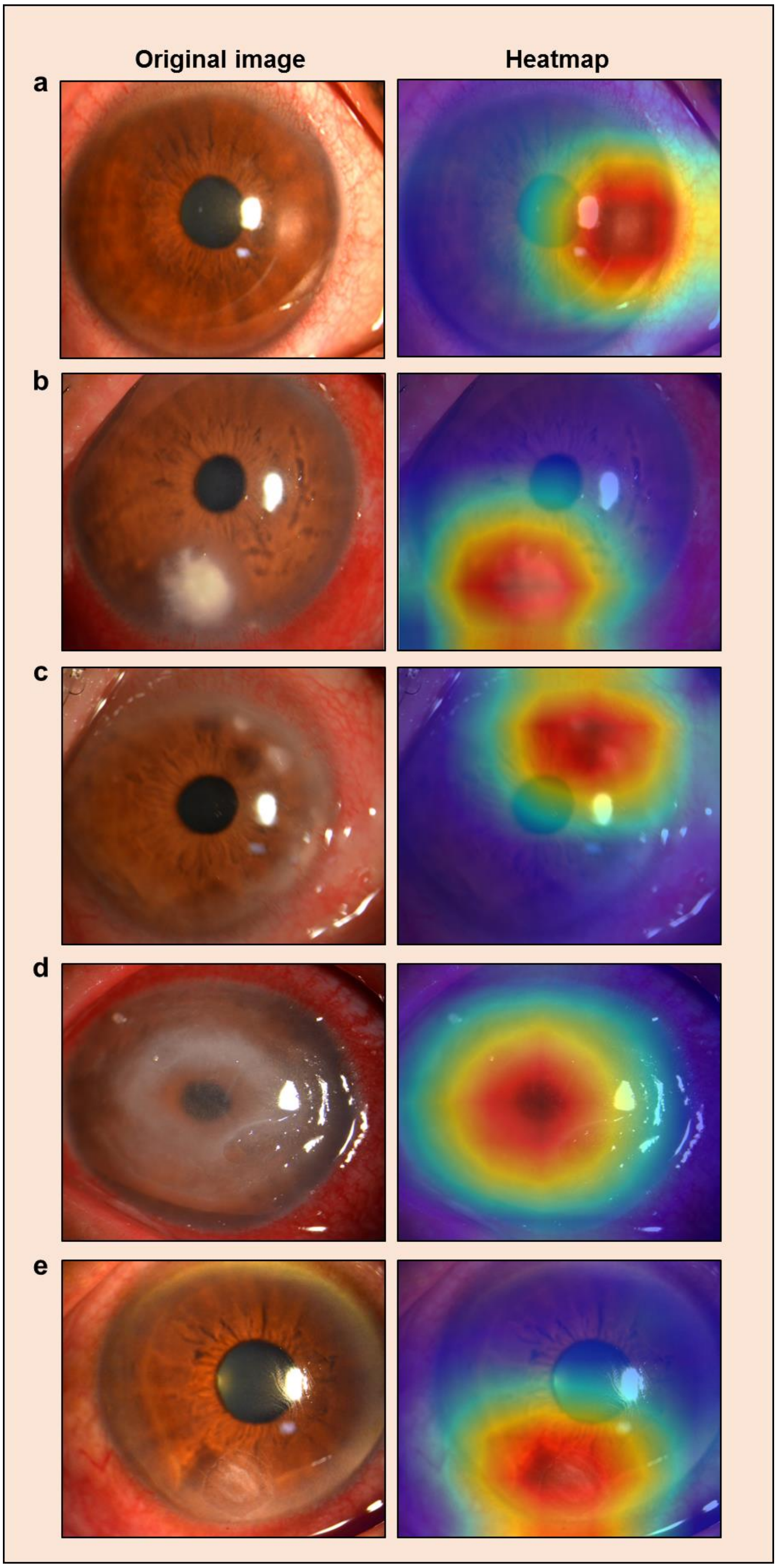

Supplementary Figure 18. Gradient visualization of DeepIK diagnoses of bacterial, fungal, viral, amebic, and noninfectious keratitis. The columns are (1) the original slit-lamp images and (2) the corresponding heatmap. The redder the area in the heatmap contributes more to the model's decision-making. **a** Bacterial keratitis. **b** Fungal keratitis. **c** Viral keratitis. **d** Amebic keratitis. **e** Noninfectious keratitis.

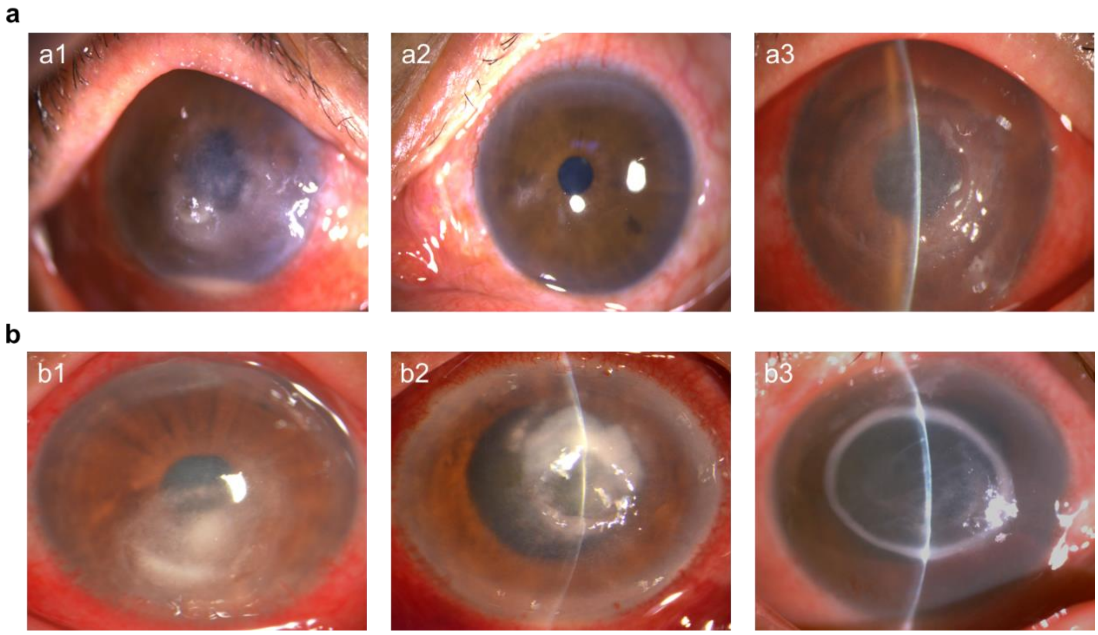

Supplementary Figure 19. Typical examples of discrepancies between DeepIK and the ophthalmologists in the contest dataset. a Typical examples that were incorrectly diagnosed by all four ophthalmologists, but correctly diagnosed by DeepIK. a1 Bacterial keratitis. a2 Fungal keratitis. a3 Amebic keratitis. **b** Typical examples that were correctly diagnosed by all four ophthalmologists, but incorrectly diagnosed by DeepIK. b1 Bacterial keratitis. b2 Fungal keratitis. b3 Amebic keratitis.

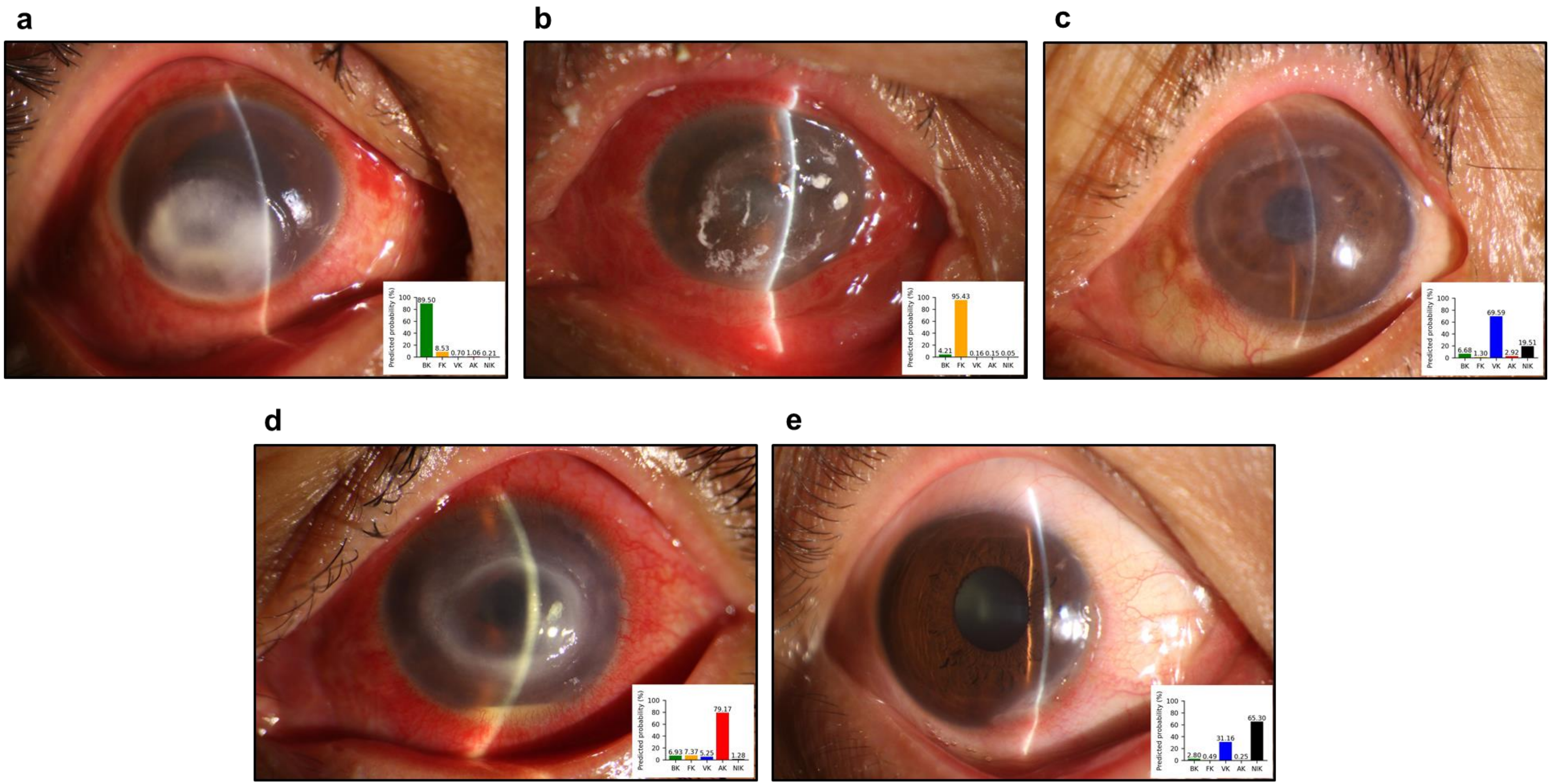

Supplementary Figure 20. Representative examples of images with predicted probability provided by DeepIK. The predicted probability map for each type of keratitis is located in the lower right of the slit lamp image. **a** bacterial keratitis. **b** Fungal keratitis. **c** Viral keratitis. **d** Amebic keratitis. **e** Noninfectious keratitis (peripheral ulcerative keratitis).

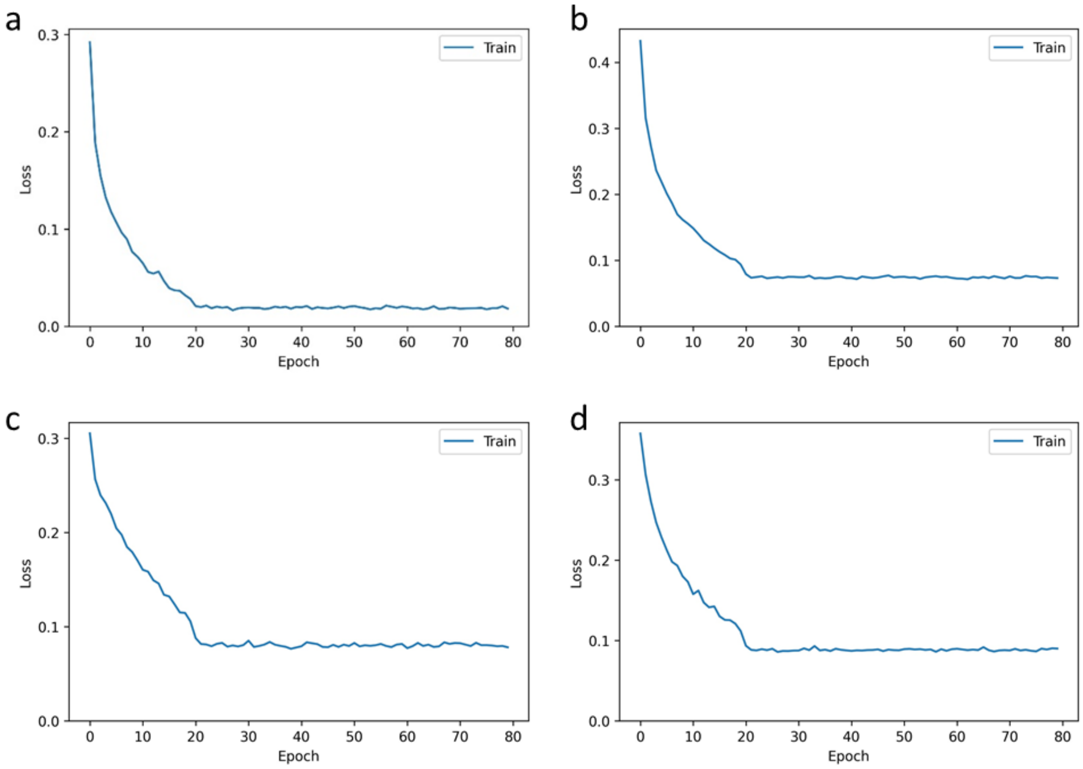

Supplementary Figure 21. Loss curves during deep learning model training. a Loss curve during the training process of DeepIK. **b** Loss curve during the training process of DenseNet121. **c** Loss curve during the training process of InceptionResNetV2. **d** Loss curve during the training process of Swin-Transformer.

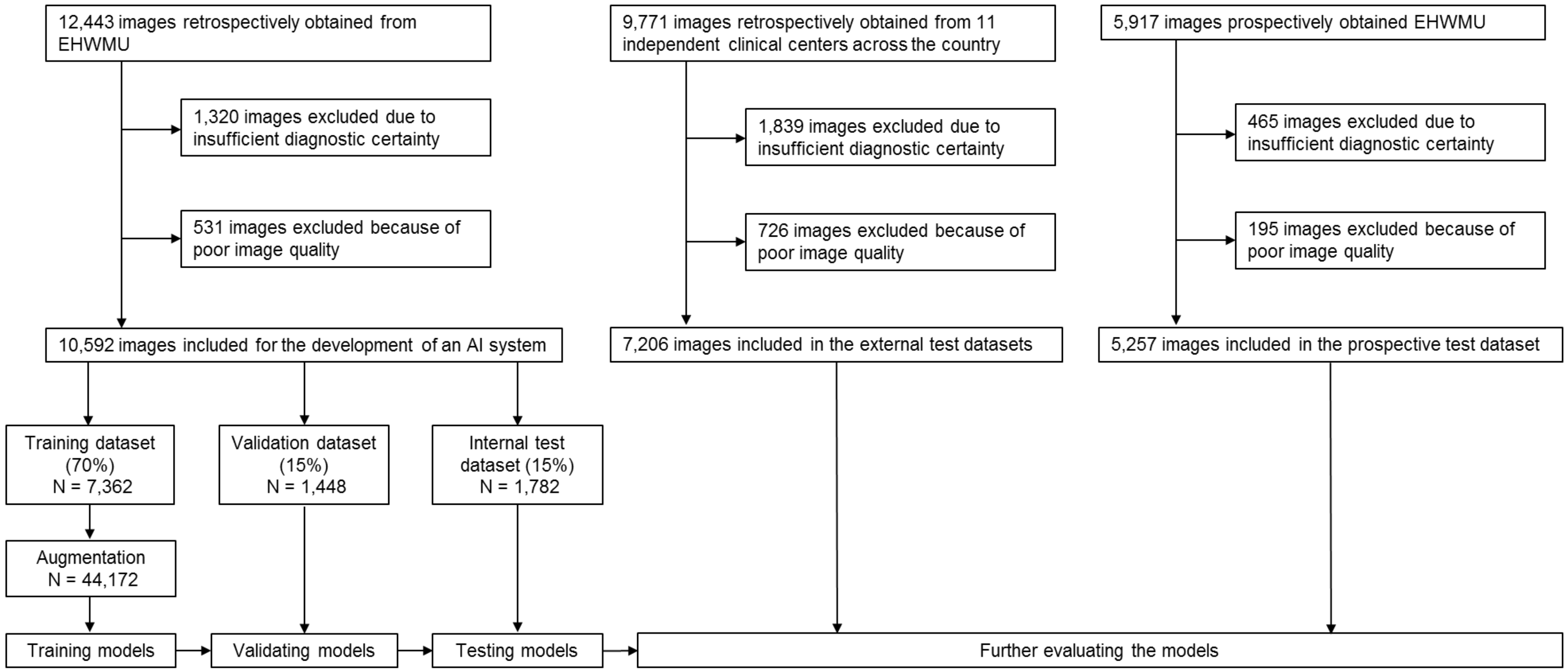

Supplementary Figure 22. Workflow diagram of the development and evaluation of deep learning models. AI, artificial intelligence. EHWMU, Eye Hospital of Wenzhou Medical University.

Supplementary Table 1. Demographic characteristics of patients from the development dataset and the external and prospective test datasets.

Clinical center	Location (City, Province)	Number of patients	Age, mean/range (years)	No. (%) of women
Development dataset				
EHWMU	Wenzhou, Zhejiang	6,300	51.8 (0.33-100)	2,785 (44.2)
External test datasets				
NEH	Ningbo, Zhejiang	134	55.1 (4-89)	41 (30.6)
DNPH	Dalian, Liaoning	406	59.8 (6-90)	140 (34.4)
FAHFMU	Fuzhou, Fujian	216	55.5 (3-89)	82 (37.9)
SHLU	Lanzhou, Gansu	57	54.1 (1-78)	26 (45.6)
AEHNU	Nanchang, Jiangxi	456	52.5 (1-92)	146 (32.0)
AEHNMU	Nanjing, Jiangsu	437	55.7 (2-94)	135 (30.8%)
PHNHAR	Yinchuan, Ningxia	317	52.3 (2-88)	138 (43.5)
RHWU	Wuhan, Hubei	257	55.1 (0.25-88)	115 (44.7)
XNH	Xi'an, Shaanxi	603	57.4 (2-84)	232 (38.4)
TXHCSU	Changsha, Hunan	224	49.3 (4-84)	102 (45.5)
AHGMU	Guiyang, Guizhou	69	46.1 (19-82)	21 (30.4)
Prospective test dataset				
EHWMU	Wenzhou, Zhejiang	893	52.6 (4-90)	371 (41.5)
Total at all centers		10,369	53.6 (0.25-100)	4,334 (41.8)

EHWMU, Eye Hospital of Wenzhou Medical University. NEH, Ningbo Eye Hospital. DNPH, Dalian No.3 People's Hospital. FAHFMU, First Affiliated Hospital of Fujian Medical University. SHLU, Second Hospital of Lanzhou University. AEHNU, Affiliated Eye Hospital of Nanchang University. AEHNMU, Affiliated Eye Hospital of Nanjing Medical University. PHNHAR, People's Hospital of Ningxia Hui Autonomous Region. RHWU, Renmin Hospital of Wuhan University. XNH, Xi'an No.1 Hospital. TXHCSU, Third Xiangya Hospital of Central South University. AHGMU, Affiliated Hospital of Guizhou Medical University.

Supplementary Table 2. Performance of deep learning algorithms in an internal test dataset.

One-vs.-rest classification	EHWMU internal test dataset		
	**Accuracy (95% CI)**	**Sensitivity (95% CI)**	**Specificity (95% CI)**
**BK vs. FK + VK + AK + NIK**			
DeepIK	91.3% (90.0-92.6)	76.9% (71.8-82.0)	93.8% (92.5-95.0)
DenseNet121	88.8% (87.3-90.2)	69.2% (63.6-74.8)	92.1% (90.8-93.5)
InceptionResNetV2	89.3% (87.8-90.7)	53.1% (47.0-59.1)	95.5% (94.4-96.5)
Swin-Transformer	88.0% (86.5-99.5)	69.2% (63.6-74.8)	91.2% (89.8-92.6)
**FK vs. BK + VK + AK + NIK**			
DeepIK	94.1% (93.0-95.1)	79.7% (74.9-84.5)	96.6% (95.7-97.5)
DenseNet121	92.5% (91.3-93.8)	68.3% (62.7-73.8)	96.9% (96.0-97.8)
InceptionResNetV2	89.2% (87.7-90.6)	73.1% (67.8-78.3)	92.1% (90.7-93.4)
Swin-Transformer	93.0% (91.9-94.2)	64.6% (58.9-70.3)	98.1% (97.5-98.8)
**VK vs. BK + FK + AK + NIK**			
DeepIK	88.7% (87.3-90.2)	83.5% (80.6-86.3)	91.7% (90.1-93.3)
DenseNet121	88.5% (87.0-90.0)	83.6% (80.8-86.5)	91.3% (89.6-92.9)
InceptionResNetV2	84.4% (82.7-86.1)	80.7% (77.6-83.7)	86.5% (84.5-88.5)
Swin-Transformer	87.6% (86.1-89.1)	84.5% (81.8-87.3)	89.3% (87.5-91.1)
**AK vs. BK + FK + VK + NIK**			
DeepIK	98.9% (98.5-99.4)	75.0% (65.0-85.0)	99.9% (99.8-100)
DenseNet121	98.6% (98.1-99.1)	77.8% (68.2-87.4)	99.5% (99.1-99.8)
InceptionResNetV2	97.2% (96.4-98.0)	83.3% (74.7-91.9)	97.8% (97.1-98.5)
Swin-Transformer	98.3% (97.7-98.9)	58.3% (46.9-69.7)	99.9% (99.8-100)
**NIK vs. BK + FK + VK + AK**			
DeepIK	93.7% (92.5-94.8)	89.3% (86.7-91.9)	95.5% (94.4-96.7)
DenseNet121	93.1% (91.9-94.3)	89.7% (87.1-92.2)	94.6% (93.3-95.8)
InceptionResNetV2	92.5% (91.3-93.7)	82.9% (79.7-86.1)	96.6% (95.5-97.6)
Swin-Transformer	91.9% (90.7-93.2)	88.5% (95.8-91.2)	93.4% (92.0-94.7)

EHWMU, Eye Hospital of Wenzhou Medical University. CI, confidence interval. BK, bacterial keratitis. FK, fungal keratitis. VK, viral keratitis. AK, amebic keratitis. NIK, noninfectious keratitis.

Supplementary Table 3. Performance of deep learning algorithms in external test datasets.

One-vs.-rest classification	External test datasets[a]		
	**Accuracy (95% CI)**	**Sensitivity (95% CI)**	**Specificity (95% CI)**
**BK vs. FK + VK + AK + NIK**			
DeepIK	88.9% (88.1-89.6)	74.8% (72.6-77.0)	92.7% (92.0-93.4)
DenseNet121	73.6% (72.6-74.6)	73.6% (71.4-75.8)	73.6% (72.4-74.7)
InceptionResNetV2	73.3% (72.3-74.4)	39.6% (37.2-42.0)	82.6% (81.6-83.6)
Swin-Transformer	69.8% (68.7-70.9)	68.3% (66.0-70.6)	70.2% (69.0-71.4)
**FK vs. BK + VK + AK + NIK**			
DeepIK	91.5% (90.8-92.1)	80.5% (78.7-82.3)	95.3% (94.7-95.9)
DenseNet121	82.6% (81.7-83.5)	50.2% (47.9-52.5)	93.9% (93.3-94.6)
InceptionResNetV2	70.1% (69.1-71.2)	42.6% (40.4-44.9)	79.7% (78.6-80.8)
Swin-Transformer	80.8% (79.9-81.7)	33.2% (31.1-35.3)	97.5% (97.1-97.9)
**VK vs. BK + FK + AK + NIK**			
DeepIK	88.9% (88.2-89.6)	80.5% (78.9-82.2)	92.6% (91.8-93.3)
DenseNet121	78.4% (77.4-79.3)	53.3% (51.2-55.4)	89.3% (88.4-90.1)
InceptionResNetV2	69.7% (68.7-70.8)	54.6% (52.5-56.7)	76.4% (75.2-77.5)
Swin-Transformer	76.5% (75.5-77.4)	49.2% (47.1-51.3)	88.3% (87.5-89.2)
**AK vs. BK + FK + VK + NIK**			
DeepIK	99.0% (98.7-99.2)	68.3% (59.3-77.2)	99.4% (99.2-99.6)
DenseNet121	98.4% (98.1-98.7)	23.1% (15.0-31.2)	99.5% (99.4-99.7)
InceptionResNetV2	93.4% (92.8-94.0)	26.9% (18.4-35.4)	94.4% (93.8-94.9)
Swin-Transformer	98.4% (98.1-98.7)	14.4% (7.7-21.2)	99.6% (99.5-99.8)
**NIK vs. BK + FK + VK + AK**			
DeepIK	90.3% (89.6-91.0)	81.1% (79.1-83.1)	92.7% (92.0-93.4)
DenseNet121	81.8% (80.9-82.7)	57.8% (55.3-60.3)	88.1% (87.2-88.9)
InceptionResNetV2	77.1% (76.2-78.1)	25.8% (23.6-28.1)	90.6% (89.9-91.4)
Swin-Transformer	78.7% (77.8-79.7)	65.8% (63.4-68.2)	82.1% (81.1-83.1)

[a]External test datasets include images collected from 11 independent clinical centers. CI, confidence interval. BK, bacterial keratitis. FK, fungal keratitis. VK, viral keratitis. AK, amebic keratitis. NIK, noninfectious keratitis.

Supplementary Table 4. Performance of deep learning algorithms in the NEH external test dataset.

One-vs.-rest classification	NEH external test dataset		
	**Accuracy (95% CI)**	**Sensitivity (95% CI)**	**Specificity (95% CI)**
**BK vs. FK + VK + NIK**			
DeepIK	88.4% (85.6-91.3)	72.1% (58.7-85.5)	90.1% (87.3-92.9)
DenseNet121	80.7% (77.1-84.2)	74.4% (61.4-87.5)	81.3% (77.6-85.0)
InceptionResNetV2	80.7% (77.1-84.2)	20.9% (8.8-33.1)	86.6% (83.4-89.8)
Swin-Transformer	73.1% (69.1-77.1)	81.4% (69.8-93.0)	72.3% (68.1-76.5)
**FK vs. BK + VK + NIK**			
DeepIK	91.4% (88.9-93.9)	84.0% (77.7-90.3)	94.2% (91.7-96.7)
DenseNet121	82.8% (79.4-86.2)	65.6% (57.5-73.8)	89.3% (86.0-92.5)
InceptionResNetV2	68.5% (64.3-72.7)	53.4% (44.9-62.0)	74.2% (69.6-78.8)
Swin-Transformer	80.3% (76.7-83.8)	44.3% (35.8-52.8)	93.9% (91.4-96.4)
**VK vs. BK + FK + NIK**			
DeepIK	89.5% (86.7-92.3)	77.1% (70.7-83.4)	96.4% (94.3-98.5)
DenseNet121	82.1% (78.7-85.6)	71.8% (65.0-78.5)	87.9% (84.3-91.6)
InceptionResNetV2	72.7% (68.7-76.7)	58.8% (51.4-66.2)	80.4% (75.9-84.8)
Swin-Transformer	85.5% (82.3-88.7)	70.0% (63.1-76.9)	94.1% (91.5-96.8)
**NIK vs. BK + FK + VK**			
DeepIK	90.1% (87.4-92.8)	80.3% (73.5-87.1)	93.9% (91.4-96.4)
DenseNet121	80.5% (76.9-84.0)	45.5% (37.0-53.9)	93.9% (91.4-96.4)
InceptionResNetV2	72.5% (68.5-76.5)	15.2% (9.0-21.3)	94.5% (92.1-96.9)
Swin-Transformer	79.2% (75.6-82.8)	52.3% (43.8-60.8)	89.5% (86.3-92.8)

NEH, Ningbo Eye Hospital. CI, confidence interval. BK, bacterial keratitis. FK, fungal keratitis. VK, viral keratitis. NIK, noninfectious keratitis.

Supplementary Table 5. Performance of deep learning algorithms in the DNPH external test dataset.

One-vs.-rest classification	DNPH external test dataset		
	**Accuracy (95% CI)**	**Sensitivity (95% CI)**	**Specificity (95% CI)**
**BK vs. FK + VK + NIK**			
DeepIK	94.3% (92.9-95.8)	78.9% (71.5-86.4)	96.4% (95.2-97.7)
DenseNet121	75.5% (72.8-78.2)	85.1% (78.5-91.6)	74.2% (71.2-77.2)
InceptionResNetV2	68.7% (65.7-71.6)	25.4% (17.4-33.4)	74.6% (71.6-77.5)
Swin-Transformer	64.2% (61.2-67.3)	82.5% (75.5-89.4)	61.8% (58.5-65.1)
**FK vs. BK + VK + NIK**			
DeepIK	90.3% (88.4-92.2)	84.1% (80.8-87.4)	96.5% (94.8-98.1)
DenseNet121	76.9% (74.2-79.5)	62.3% (57.9-66.7)	91.2% (88.7-93.8)
InceptionResNetV2	59.0% (55.9-62.1)	34.5% (30.2-38.8)	83.1% (79.7-86.4)
Swin-Transformer	64.2% (61.2-67.3)	28.8% (24.7-32.9)	99.2% (98.3-100)
**VK vs. BK + FK + NIK**			
DeepIK	94.0% (92.5-95.5)	86.1% (81.8-90.5)	96.7% (95.4-98.1)
DenseNet121	80.5% (78.0-83.1)	30.6% (24.8-36.4)	97.9% (96.8-98.9)
InceptionResNetV2	76.3% (73.6-79.0)	35.9% (29.9-41.9)	90.4% (88.2-92.5)
Swin-Transformer	79.4% (76.8-82.0)	23.7% (18.4-29.0)	98.7% (97.9-99.6)
**NIK vs. BK + FK + VK**			
DeepIK	91.2% (89.4-93.0)	90.8% (85.7-96.0)	91.2% (89.3-93.1)
DenseNet121	80.8% (78.3-83.3)	61.7% (53.0-70.4)	83.5% (81.0-86.0)
InceptionResNetV2	67.6% (64.6-70.6)	46.7% (37.7-55.6)	70.6% (67.5-73.7)
Swin-Transformer	70.1% (67.2-73.0)	67.5% (59.1-75.9)	70.5% (67.4-73.6)

DNPH, Dalian No.3 People's Hospital. CI, confidence interval. BK, bacterial keratitis. FK, fungal keratitis. VK, viral keratitis. NIK, noninfectious keratitis.

Supplementary Table 6. Performance of deep learning algorithms in the FAHFMU external test dataset.

One-vs.-rest classification	FAHFMU external test dataset		
	**Accuracy (95% CI)**	**Sensitivity (95% CI)**	**Specificity (95% CI)**
**BK vs. FK + VK + AK + NIK**			
DeepIK	90.2% (87.3-93.0)	76.0% (64.2-87.8)	92.1% (89.4-94.9)
DenseNet121	72.5% (68.2-76.8)	64.0% (50.7-77.3)	73.6% (69.1-78.1)
InceptionResNetV2	85.4% (82.0-88.8)	28.0% (15.6-40.4)	93.2% (90.6-95.8)
Swin-Transformer	72.5% (68.2-76.8)	76.0% (64.2-87.8)	72.0% (67.4-76.6)
**FK vs. BK + VK + AK + NIK**			
DeepIK	88.8% (85.7-91.8)	79.4% (72.3-86.4)	92.8% (89.8-95.8)
DenseNet121	78.2% (74.3-82.2)	47.6% (38.9-56.3)	91.4% (88.2-94.6)
InceptionResNetV2	65.6% (61.0-70.1)	73.0% (65.3-80.8)	62.3% (56.8-67.9)
Swin-Transformer	76.8% (72.7-80.8)	42.1% (33.4-50.7)	91.8% (88.6-94.9)
**VK vs. BK + FK + AK + NIK**			
DeepIK	89.2% (86.3-92.2)	78.6% (72.1-85.1)	95.5% (92.9-98.0)
DenseNet121	78.7% (74.8-82.6)	67.5% (60.1-74.9)	85.2% (80.9-89.5)
InceptionResNetV2	74.6% (70.5-78.8)	57.1% (49.3-65.0)	84.8% (80.5-89.2)
Swin-Transformer	78.7% (74.8-82.6)	57.8% (50.0-65.6)	90.9% (87.4-94.4)
**AK vs. BK + FK + VK + NIK**			
DeepIK	98.6% (97.4-99.7)	66.7% (35.9-97.5)	99.3% (98.4-100)
DenseNet121	98.1% (96.8-99.4)	11.1% (0-31.6)	100% (100-100)
InceptionResNetV2	92.8% (90.3-95.3)	22.2% (0-49.4)	94.4% (92.1-96.6)
Swin-Transformer	97.8% (96.5-99.2)	0% (0-0)	100% (100-100)
**NIK vs. BK + FK + VK + AK**			
DeepIK	92.6% (90.1-95.1)	86.1% (78.4-93.7)	94.1% (91.6-96.6)
DenseNet121	85.9% (82.5-89.2)	50.6% (39.6-61.7)	94.1% (91.6-96.6)
InceptionResNetV2	82.5% (78.9-86.2)	19.0% (10.3-27.6)	97.3% (95.6-99.1)
Swin-Transformer	85.6% (82.3-89.0)	67.1% (56.7-77.5)	90.0% (86.8-93.2)

FAHFMU, the First Affiliated Hospital of Fujian Medical University. CI, confidence interval. BK, bacterial keratitis. FK, fungal keratitis. VK, viral keratitis. AK, amebic keratitis. NIK, noninfectious keratitis.

Supplementary Table 7. Performance of deep learning algorithms in the SHLU external test dataset.

One-vs.-rest classification	SHLU external test dataset		
	**Accuracy (95% CI)**	**Sensitivity (95% CI)**	**Specificity (95% CI)**
**BK vs. FK + VK + AK + NIK**			
DeepIK	80.5% (75.6-85.3)	72.9% (64.9-80.9)	86.7% (81.1-92.3)
DenseNet121	67.0% (61.3-72.8)	75.4% (67.7-83.2)	60.1% (52.1-68.2)
InceptionResNetV2	51.7% (45.7-57.8)	33.1% (24.6-41.5)	67.1% (59.4-74.8)
Swin-Transformer	58.6% (52.6-64.6)	72.9% (64.9-80.9)	46.9% (38.7-55.0)
**FK vs. BK + VK + AK + NIK**			
DeepIK	85.8% (81.6-90.1)	80.8% (72.0-89.5)	88.0% (83.3-92.7)
DenseNet121	78.2% (73.1-83.2)	48.7% (37.6-59.8)	90.7% (86.5-94.9)
InceptionResNetV2	66.3% (60.5-72.0)	33.3% (22.9-43.8)	80.3% (74.6-86.1)
Swin-Transformer	73.6% (68.2-78.9)	23.1% (13.7-32.4)	95.1% (91.9-98.2)
**VK vs. BK + FK + AK + NIK**			
DeepIK	93.5% (90.5-96.5)	84.6% (73.3-95.9)	95.0% (92.2-97.9)
DenseNet121	83.9% (79.5-88.4)	41.0% (25.6-56.5)	91.4% (87.8-95.1)
InceptionResNetV2	73.9% (68.6-79.3)	48.7% (33.0-64.4)	78.4% (73.0-83.8)
Swin-Transformer	78.5% (73.6-83.5)	30.8% (16.3-45.3)	86.9% (82.5-91.4)
**AK vs. BK + FK + VK + NIK**			
DeepIK	98.9% (97.6-100)	0% (0-0)	98.9% (97.6-100)
DenseNet121	97.7% (95.9-99.5)	0% (0-0)	97.7% (95.9-99.5)
InceptionResNetV2	89.7% (86.0-93.3)	0% (0-0)	89.7% (86.0-93.3)
Swin-Transformer	100% (100-100)	0% (0-0)	100% (100-100)
**NIK vs. BK + FK + VK + AK**			
DeepIK	95.4% (92.9-97.9)	73.1% (56.0-90.1)	97.9% (96.0-99.7)
DenseNet121	94.3% (91.4-97.1)	57.7% (38.7-76.7)	98.3% (96.6-100)
InceptionResNetV2	86.6% (82.5-90.7)	19.2% (4.1-34.4)	94.0% (91.0-97.1)
Swin-Transformer	89.7% (86.0-93.3)	57.7% (38.7-76.7)	93.2% (90.0-96.4)

SHLU, Second Hospital of Lanzhou University. CI, confidence interval. BK, bacterial keratitis. FK, fungal keratitis. VK, viral keratitis. AK, amebic keratitis. NIK, noninfectious keratitis.

Supplementary Table 8. Performance of deep learning algorithms in the AEHNU external test dataset.

One-vs.-rest classification	AEHNU external test dataset		
	**Accuracy (95% CI)**	**Sensitivity (95% CI)**	**Specificity (95% CI)**
**BK vs. FK + VK + AK + NIK**			
DeepIK	89.9% (87.2-92.6)	74.5% (62.5-86.5)	91.8% (89.2-94.4)
DenseNet121	69.7% (65.6-73.9)	54.9% (41.2-68.6)	71.5% (67.2-75.8)
InceptionResNetV2	74.6% (70.7-78.5)	41.2% (27.7-54.7)	78.6% (74.7-82.5)
Swin-Transformer	67.4% (63.2-71.6)	60.8% (47.4-74.2)	68.2% (63.8-72.7)
**FK vs. BK + VK + AK + NIK**			
DeepIK	91.2% (88.6-93.7)	81.7% (76.0-87.3)	97.0% (95.0-98.9)
DenseNet121	78.8% (75.1-82.5)	50.0% (42.7-57.3)	96.3% (94.1-98.4)
InceptionResNetV2	72.3% (68.2-76.3)	39.4% (32.3-46.6)	92.2% (89.2-95.3)
Swin-Transformer	73.9% (70.0-77.9)	32.2% (25.4-39.0)	99.3% (98.4-100)
**VK vs. BK + FK + AK + NIK**			
DeepIK	88.7% (85.8-91.5)	82.3% (76.3-88.2)	91.8% (88.8-94.8)
DenseNet121	77.3% (73.5-81.1)	58.9% (51.2-66.5)	86.5% (82.7-90.2)
InceptionResNetV2	63.9% (59.5-68.2)	67.1% (59.8-74.4)	62.3% (56.9-67.6)
Swin-Transformer	72.5% (68.5-76.5)	54.4% (46.7-62.2)	81.4% (77.2-85.7)
**AK vs. BK + FK + VK + NIK**			
DeepIK	97.7% (96.3-99.0)	70.0% (53.6-86.4)	99.6% (98.9-100)
DenseNet121	95.8% (94.0-97.6)	36.7% (19.4-53.9)	99.8% (99.3-100)
InceptionResNetV2	91.2% (88.6-93.7)	13.3% (1.2-25.5)	96.4% (94.7-98.1)
Swin-Transformer	95.4% (93.5-97.3)	26.7% (10.8-42.5)	100% (100-100)
**NIK vs. BK + FK + VK + AK**			
DeepIK	93.1% (90.8-95.3)	80.7% (70.5-90.9)	94.7% (92.6-96.9)
DenseNet121	85.5% (82.3-88.7)	57.9% (45.1-70.7)	89.3% (86.3-92.2)
InceptionResNetV2	86.3% (83.3-89.4)	14.0% (5.0-23.1)	96.2% (94.3-98.0)
Swin-Transformer	83.2% (79.8-86.6)	64.9% (52.5-77.3)	85.7% (82.3-89.0)

AEHNU, Affiliated Eye Hospital of Nanchang University. CI, confidence interval. BK, bacterial keratitis. FK, fungal keratitis. VK, viral keratitis. AK, amebic keratitis. NIK, noninfectious keratitis.

Supplementary Table 9. Performance of deep learning algorithms in the AEHNMU external test dataset.

One-vs.-rest classification	AEHNMU external test dataset		
	**Accuracy (95% CI)**	**Sensitivity (95% CI)**	**Specificity (95% CI)**
**BK vs. FK + VK + AK + NIK**			
DeepIK	85.1% (81.1-89.0)	78.0% (70.7-85.4)	89.7% (85.4-94.1)
DenseNet121	71.8% (66.7-76.8)	75.6% (68.0-83.2)	69.2% (62.5-75.8)
InceptionResNetV2	60.4% (54.9-65.9)	29.3% (21.2-37.3)	81.1% (75.4-86.7)
Swin-Transformer	66.2% (61.0-71.5)	78.0% (70.7-85.4)	58.4% (51.3-65.5)
**FK vs. BK + VK + AK + NIK**			
DeepIK	86.0% (82.2-89.9)	84.5% (77.5-91.5)	86.8% (82.2-91.5)
DenseNet121	80.2% (75.7-84.6)	67.0% (57.9-76.1)	86.8% (82.2-91.5)
InceptionResNetV2	67.5% (62.3-72.8)	62.1% (52.8-71.5)	70.2% (64.0-76.5)
Swin-Transformer	78.2% (73.6-82.9)	44.7% (35.1-54.3)	95.1% (92.2-98.1)
**VK vs. BK + FK + AK + NIK**			
DeepIK	94.5% (91.9-97.0)	77.8% (62.1-93.5)	96.1% (93.8-98.4)
DenseNet121	90.3% (86.9-93.6)	63.0% (44.7-81.2)	92.9% (89.9-95.9)
InceptionResNetV2	89.9% (86.6-93.3)	37.0% (18.8-55.3)	95.0% (92.5-97.6)
Swin-Transformer	88.0% (84.4-91.6)	74.1% (57.5-90.6)	89.3% (85.7-92.9)
**AK vs. BK + FK + VK + NIK**			
DeepIK	95.5% (93.1-97.8)	64.3% (46.5-82.0)	98.6% (97.2-100)
DenseNet121	91.6% (88.5-94.7)	10.7% (0-22.2)	99.6% (98.9-100)
InceptionResNetV2	74.0% (69.1-78.9)	35.7% (18.0-53.5)	77.9% (73.0-82.7)
Swin-Transformer	90.0% (87.7-94.1)	10.7% (0-22.2)	98.9% (97.7-100)
**NIK vs. BK + FK + VK + AK**			
DeepIK	96.1% (93.9-98.3)	74.1% (57.5-90.6)	98.2% (96.7-99.8)
DenseNet121	94.8% (92.3-97.3)	59.3% (40.7-77.8)	98.2% (96.7-99.8)
InceptionResNetV2	93.8% (91.1-96.5)	44.4% (25.7-63.2)	98.6% (97.2-100)
Swin-Transformer	94.8% (92.3-97.3)	63.0% (44.7-81.2)	97.9% (96.2-99.6)

AEHNMU, Affiliated Eye Hospital of Nanjing Medical University. CI, confidence interval. BK, bacterial keratitis. FK, fungal keratitis. VK, viral keratitis. AK, amebic keratitis. NIK, noninfectious keratitis.

Supplementary Table 10. Performance of deep learning algorithms in the PHNHAR external test dataset.

One-vs.-rest classification	PHNHAR external test dataset		
	**Accuracy (95% CI)**	**Sensitivity (95% CI)**	**Specificity (95% CI)**
**BK vs. FK + VK + NIK**			
DeepIK	89.4% (87.3-91.4)	77.8% (72.7-82.8)	94.3% (92.5-96.1)
DenseNet121	75.9% (73.1-78.7)	65.1% (59.4-70.9)	80.5% (77.4-83.6)
InceptionResNetV2	70.8% (67.8-73.8)	10.3% (6.7-14.0)	96.4% (95.0-97.9)
Swin-Transformer	73.2% (70.2-76.1)	67.0% (61.3-72.8)	75.8% (72.4-79.2)
**FK vs. BK + VK + NIK**			
DeepIK	93.0% (91.4-94.7)	80.8% (74.0-87.5)	95.2% (93.6-96.7)
DenseNet121	89.3% (87.2-91.3)	50.0% (41.4-58.6)	96.1% (94.7-97.5)
InceptionResNetV2	65.6% (62.5-68.8)	57.7% (49.2-66.2)	67.0% (63.7-70.4)
Swin-Transformer	89.5% (87.5-91.5)	40.8% (32.3-49.2)	98.0% (97.0-99.0)
**VK vs. BK + FK + NIK**			
DeepIK	87.2% (85.0-89.4)	85.2% (80.5-89.9)	87.9% (85.4-90.4)
DenseNet121	76.6% (73.8-79.4)	68.6% (62.5-74.7)	79.3% (76.2-82.4)
InceptionResNetV2	51.1% (47.8-54.5)	54.7% (48.2-61.2)	49.9% (46.1-53.8)
Swin-Transformer	77.9% (75.1-80.6)	57.4% (50.9-63.9)	84.8% (82.1-87.6)
**NIK vs. BK + FK + VK**			
DeepIK	91.9% (90.1-93.7)	79.8% (74.9-84.6)	97.1% (95.7-98.4)
DenseNet121	84.1% (81.7-86.6)	61.1% (55.2-67.0)	94.0% (92.1-95.9)
InceptionResNetV2	70.4% (67.4-73.5)	8.0% (4.7-11.3)	97.1% (95.7-98.4)
Swin-Transformer	84.7% (82.3-87.1)	72.1% (66.7-77.6)	90.1% (87.7-92.4)

PHNHAR, People's Hospital of Ningxia Hui Autonomous Region. CI, confidence interval. BK, bacterial keratitis. FK, fungal keratitis. VK, viral keratitis. NIK, noninfectious keratitis.

Supplementary Table 11. Performance of deep learning algorithms in the RHWU external test dataset.

One-vs.-rest classification	RHWU external test dataset		
	**Accuracy (95% CI)**	**Sensitivity (95% CI)**	**Specificity (95% CI)**
**BK vs. FK + VK + NIK**			
DeepIK	86.2% (84.2-88.2)	74.1% (68.8-79.3)	89.9% (87.9-91.9)
DenseNet121	70.6% (67.9-73.2)	79.7% (74.9-84.5)	67.8% (64.7-70.9)
InceptionResNetV2	73.0% (70.4-75.6)	52.3% (46.3-58.3)	79.4% (76.7-82.0)
Swin-Transformer	66.6% (63.9-69.3)	78.2% (73.2-83.2)	63.1% (59.9-66.3)
**FK vs. BK + VK + NIK**			
DeepIK	89.6% (87.9-91.4)	77.3% (72.8-81.8)	94.7% (93.1-96.2)
DenseNet121	79.8% (77.5-82.1)	38.5% (33.2-43.7)	96.7% (95.4-97.9)
InceptionResNetV2	72.4% (69.8-75.0)	28.2% (23.3-33.0)	90.5% (88.4-92.5)
Swin-Transformer	80.7% (78.4-83.0)	39.1% (33.8-44.4)	97.6% (96.6-98.7)
**VK vs. BK + FK + NIK**			
DeepIK	89.3% (87.5-91.1)	78.6% (74.5-82.8)	94.5% (92.9-96.1)
DenseNet121	82.7% (80.5-84.9)	63.1% (58.2-68.0)	92.3% (90.4-94.2)
InceptionResNetV2	75.6% (73.1-78.1)	71.1% (66.5-75.7)	77.7% (74.8-80.7)
Swin-Transformer	78.8% (76.4-81.2)	49.7% (44.7-54.8)	93.1% (91.3-94.9)
**NIK vs. BK + FK + VK**			
DeepIK	91.5% (89.9-93.1)	82.7% (77.0-88.5)	93.0% (91.4-94.6)
DenseNet121	82.9% (80.7-85.1)	49.4% (41.8-57.0)	88.7% (86.7-90.7)
InceptionResNetV2	83.1% (81.0-85.3)	18.5% (12.6-24.3)	94.3% (92.9-95.8)
Swin-Transformer	81.0% (78.7-83.3)	50.6% (43.0-58.2)	86.3% (84.1-88.5)

RHWU, Renmin Hospital of Wuhan University. CI, confidence interval. BK, bacterial keratitis. FK, fungal keratitis. VK, viral keratitis. NIK, noninfectious keratitis.

Supplementary Table 12. Performance of deep learning algorithms in the XNH external test dataset.

One-vs.-rest classification	XNH external test dataset		
	**Accuracy (95% CI)**	**Sensitivity (95% CI)**	**Specificity (95% CI)**
**BK vs. FK + VK + AK + NIK**			
DeepIK	85.5% (83.4-87.5)	70.8% (66.2-75.4)	93.0% (91.1-94.8)
DenseNet121	72.1% (69.5-74.8)	75.6% (71.2-80.0)	70.3% (67.0-73.6)
InceptionResNetV2	71.0% (68.3-73.7)	64.9% (60.0-69.7)	74.2% (71.0-77.4)
Swin-Transformer	69.4% (66.7-72.1)	51.5% (46.4-56.5)	78.6% (75.6-81.6)
**FK vs. BK + VK + AK + NIK**			
DeepIK	96.6% (95.6-97.7)	76.1% (68.1-84.1)	98.9% (98.2-99.5)
DenseNet121	91.6% (90.0-93.3)	31.2% (22.5-39.9)	98.3% (97.5-99.1)
InceptionResNetV2	86.4% (84.3-88.4)	22.0% (14.2-29.8)	93.4% (91.9-95.0)
Swin-Transformer	91.7% (90.1-93.4)	19.3% (11.9-26.7)	99.7% (99.4-100)
**VK vs. BK + FK + AK + NIK**			
DeepIK	85.3% (83.2-87.4)	75.7% (70.7-80.6)	88.7% (86.5-90.9)
DenseNet121	76.3% (73.8-78.8)	40.6% (35.0-46.3)	88.9% (86.8-91.1)
InceptionResNetV2	73.2% (70.6-75.8)	56.9% (51.2-62.7)	79.0% (76.2-81.8)
Swin-Transformer	70.8% (68.2-73.5)	44.4% (38.7-50.2)	80.2% (77.5-82.9)
**AK vs. BK + FK + VK + NIK**			
DeepIK	99.1% (98.5-99.7)	71.4% (52.1-90.8)	99.6% (99.3-100)
DenseNet121	98.5% (97.7-99.2)	28.6% (9.2-47.9)	99.8% (99.6-100)
InceptionResNetV2	94.4% (93.0-95.7)	38.1% (17.3-58.9)	95.5% (94.2-96.7)
Swin-Transformer	97.9% (97.1-98.8)	9.5% (0-22.1)	99.6% (99.3-100)
**NIK vs. BK + FK + VK + AK**			
DeepIK	83.4% (81.2-85.6)	79.0% (74.5-83.6)	85.1% (82.6-87.6)
DenseNet121	75.7% (73.2-78.3)	61.3% (55.9-66.7)	81.4% (78.7-84.1)
InceptionResNetV2	74.4% (71.8-77.0)	35.2% (29.8-40.5)	89.8% (87.6-91.9)
Swin-Transformer	73.5% (70.9-76.1)	72.9% (68.0-77.9)	73.7% (70.6-76.8)

XNH, Xi'an No.1 Hospital. CI, confidence interval. BK, bacterial keratitis. FK, fungal keratitis. VK, viral keratitis. AK, amebic keratitis. NIK, noninfectious keratitis.

Supplementary Table 13. Performance of deep learning algorithms in the TXHCSU external test dataset.

One-vs.-rest classification	TXHCSU external test dataset		
	**Accuracy (95% CI)**	**Sensitivity (95% CI)**	**Specificity (95% CI)**
**BK vs. FK + VK + NIK**			
DeepIK	95.0% (93.4-96.5)	78.9% (69.4-88.4)	96.5% (95.2-97.9)
DenseNet121	79.6% (76.8-82.4)	67.6% (56.7-78.5)	80.7% (77.9-83.6)
InceptionResNetV2	79.9% (77.2-82.7)	25.4% (15.2-35.5)	85.3% (82.7-87.9)
Swin-Transformer	80.7% (78.0-83.5)	54.9% (43.4-66.5)	83.2% (80.5-86.0)
**FK vs. BK + VK + NIK**			
DeepIK	92.3% (90.5-94.2)	73.3% (65.4-81.2)	95.7% (94.2-97.2)
DenseNet121	80.6% (77.8-83.3)	34.2% (25.7-42.7)	88.9% (86.5-91.2)
InceptionResNetV2	66.1% (62.8-69.4)	65.8% (57.3-74.3)	66.1% (62.5-69.7)
Swin-Transformer	86.0% (83.6-88.4)	23.3% (15.8-30.9)	97.2% (95.9-98.4)
**VK vs. BK + FK + NIK**			
DeepIK	84.2% (81.7-86.8)	82.0% (78.4-85.6)	86.8% (83.4-90.3)
DenseNet121	66.8% (63.6-70.1)	46.0% (41.3-50.8)	91.2% (88.3-94.1)
InceptionResNetV2	58.5% (55.1-61.9)	40.9% (36.2-45.5)	79.2% (75.0-83.3)
Swin-Transformer	64.7% (61.4-68.0)	47.4% (42.7-52.2)	84.9% (81.3-88.6)
**NIK vs. BK + FK + VK**			
DeepIK	88.7% (86.4-90.9)	79.3% (73.3-85.3)	91.3% (89.1-93.5)
DenseNet121	75.3% (72.3-78.3)	66.7% (59.7-73.7)	77.7% (74.4-81.0)
InceptionResNetV2	75.0% (72.0-78.0)	22.4% (16.2-28.6)	89.8% (87.4-92.2)
Swin-Transformer	70.0% (6.68-73.2)	75.3% (68.9-81.7)	68.5% (64.8-72.2)

TXHCSU, Third Xiangya Hospital of Central South University. CI, confidence interval. BK, bacterial keratitis. FK, fungal keratitis. VK, viral keratitis. NIK, noninfectious keratitis.

Supplementary Table 14. Performance of deep learning algorithms in the AHGMU external test dataset.

One-vs.-rest classification	AHGMU external test dataset		
	**Accuracy (95% CI)**	**Sensitivity (95% CI)**	**Specificity (95% CI)**
**BK vs. FK + VK + AK + NIK**			
DeepIK	86.0% (82.7-89.4)	75.6% (66.1-85.2)	88.5% (85.0-91.9)
DenseNet121	67.6% (63.1-72.2)	71.8% (61.8-81.8)	66.7% (61.6-71.8)
InceptionResNetV2	85.3% (81.9-88.7)	50.0% (38.9-61.1)	93.6% (91.0-96.3)
Swin-Transformer	70.3% (65.9-74.8)	80.8% (72.0-89.5)	67.9% (62.8-72.9)
**FK vs. BK + VK + AK + NIK**			
DeepIK	91.2% (88.4-93.9)	77.9% (69.1-86.7)	94.7% (92.3-97.2)
DenseNet121	82.4% (78.7-86.1)	37.2% (27.0-47.4)	94.4% (91.9-96.9)
InceptionResNetV2	71.8% (67.4-76.2)	44.2% (33.7-54.7)	79.2% (74.8-83.6)
Swin-Transformer	81.4% (77.6-85.2)	22.1% (13.3-30.9)	97.2% (95.4-99.0)
**VK vs. BK + FK + AK + NIK**			
DeepIK	90.7% (87.9-93.5)	75.6% (66.3-84.9)	94.5% (92.0-97.0)
DenseNet121	76.7% (72.6-80.8)	45.1% (34.4-55.9)	84.7% (80.8-88.6)
InceptionResNetV2	71.1% (66.7-75.5)	68.3% (58.2-78.4)	71.8% (66.9-76.7)
Swin-Transformer	79.9% (76.0-83.8)	58.5% (47.9-69.2)	85.3% (81.4-89.1)
**AK vs. BK + FK + VK + NIK**			
DeepIK	97.5% (96.0-99.0)	68.8% (46.0-91.5)	98.7% (97.6-99.8)
DenseNet121	95.3% (93.3-97.4)	18.8% (0-37.9)	98.5% (97.3-99.7)
InceptionResNetV2	95.1% (93.0-97.2)	25.0% (3.8-46.2)	98.0% (96.6-99.4)
Swin-Transformer	96.1% (94.2-98.0)	12.5% (0-28.7)	99.5% (98.8-100)
**NIK vs. BK + FK + VK + AK**			
DeepIK	90.0% (87.0-92.9)	80.8% (74.4-87.2)	95.0% (92.4-97.7)
DenseNet121	79.9% (76.0-83.8)	54.8% (46.7-62.9)	93.9% (91.0-96.8)
InceptionResNetV2	79.2% (75.2-83.1)	49.3% (41.2-57.4)	95.8% (93.4-98.2)
Swin-Transformer	78.7% (74.7-82.7)	58.2% (50.2-66.2)	90.1% (86.5-93.7)

AHGMU, Affiliated Hospital of Guizhou Medical University. CI, confidence interval. BK, bacterial keratitis. FK, fungal keratitis. VK, viral keratitis. AK, amebic keratitis. NIK, noninfectious keratitis.

Supplementary Table 15. Performance of deep learning algorithms in the EHWMU prospective test dataset.

One-vs.-rest classification	EHWMU prospective test dataset		
	**Accuracy (95% CI)**	**Sensitivity (95% CI)**	**Specificity (95% CI)**
**BK vs. FK + VK + AK + NIK**			
DeepIK	93.8% (93.1-94.5)	73.8% (68.4-79.2)	94.8% (94.2-95.4)
DenseNet121	87.5% (86.6-88.4)	78.5% (73.5-83.5)	87.9% (87.0-88.8)
InceptionResNetV2	88.5% (87.7-89.4)	50.0% (43.9-56.1)	90.5% (89.7-91.3)
Swin-Transformer	87.0% (86.1-87.9)	73.0% (67.6-78.5)	87.7% (86.8-88.6)
**FK vs. BK + VK + AK + NIK**			
DeepIK	95.0% (94.4-95.6)	79.0% (76.1-82.0)	97.6% (97.2-98.1)
DenseNet121	90.9% (90.1-91.7)	44.5% (40.9-48.1)	98.5% (98.1-98.8)
InceptionResNetV2	88.2% (87.4-89.1)	48.3% (44.7-51.9)	94.7% (94.1-95.4)
Swin-Transformer	90.5% (89.7-91.3)	43.4% (39.8-47.0)	98.2% (97.8-98.6)
**VK vs. BK + FK + AK + NIK**			
DeepIK	86.2% (85.2-87.1)	82.2% (80.6-83.8)	89.2% (88.0-90.3)
DenseNet121	79.1% (78.0-80.2)	69.2% (67.3-71.1)	86.5% (85.2-87.7)
InceptionResNetV2	79.3% (78.2-80.4)	74.4% (72.6-76.2)	83.0% (81.7-84.3)
Swin-Transformer	80.1% (79.0-81.2)	71.8% (69.9-73.6)	86.3% (85.1-87.6)
**AK vs. BK + FK + VK + NIK**			
DeepIK	99.6% (99.4-99.7)	66.7% (49.8-83.5)	99.8% (99.6-99.9)
DenseNet121	99.1% (98.9-99.4)	33.3% (16.5-50.2)	99.5% (99.3-99.7)
InceptionResNetV2	96.2% (95.6-96.7)	56.7% (38.9-74.4)	96.4% (95.9-96.9)
Swin-Transformer	99.5% (99.3-99.7)	43.3% (25.6-61.1)	99.8% (99.7-100)
**NIK vs. BK + FK + VK + AK**			
DeepIK	85.1% (84.1-86.1)	78.4% (76.6-80.3)	89.1% (88.1-90.2)
DenseNet121	80.5% (79.5-81.6)	76.0% (74.1-77.9)	83.3% (82.0-84.6)
InceptionResNetV2	81.5% (80.4-82.5)	67.5% (65.4-69.5)	89.9% (88.9-91.0)
Swin-Transformer	81.4% (80.3-82.4)	75.9% (74.0-77.8)	84.7% (83.5-85.9)

EHWMU, Eye Hospital of Wenzhou Medical University. CI, confidence interval. BK, bacterial keratitis. FK, fungal keratitis. VK, viral keratitis. AK, amebic keratitis. NIK, noninfectious keratitis.

Supplementary Table 16. Comparison of DeepIK with Ophthalmologists in the classification of bacterial, fungal, viral, amebic, and noninfectious keratitis.

One-vs.-Rest Classification	DeepIK	Senior 1	Senior2	Junior1	Junior2	Junior1 + DeepIK	Junior2 + DeepIK	*P1*	*P2*	*P3*	*P4*	*P5*	*P6*
	**BK vs. FK + VK + AK + NIK**												
Accuracy	85.6%	91.2%	89.6%	77.2%	78.4%	84.8%	80.8%	0.049	0.212	0.014	0.038	0.023	0.488
(95% CI)	(81.2-90.0)	(87.7-94.7)	(85.8-93.4)	(72.0-82.4)	(73.3-83.5)	(80.3-89.3)	(75.9-85.7)						
Sensitivity	74.0%	80.0%	72.0%	54.0%	46.0%	66.0%	62.0%	0.648	1.000	0.064	0.013	0.307	0.096
(95% CI)	(61.8-86.2)	(68.9-91.1)	(59.6-84.4)	(40.2-67.8)	(32.2-59.8)	(52.9-79.1)	(48.5-75.5)						
Specificity	88.5%	94.0%	94.0%	83.0%	86.5%	89.5%	85.5%	0.043	0.071	0.126	0.636	0.053	0.864
(95% CI)	(84.1-92.9)	(90.7-97.3)	(90.7-97.3)	(77.8-88.2)	(81.8-91.2)	(85.3-93.7)	(80.6-90.4)						
	**FK vs. BK + VK + AK + NIK**												
Accuracy	91.2%	92.4%	88.8%	83.2%	84.4%	89.6%	88.4%	0.711	0.392	0.002	0.021	0.029	0.154
(95% CI)	(87.7-94.7)	(89.1-95.7)	(84.9-92.7)	(78.6-87.8)	(79.9-88.9)	(85.8-93.4)	(84.4-92.4)						
Sensitivity	78.0%	76.0%	76.0%	60.0%	52.0%	74.0%	70.0%	1.000	1.000	0.035	0.015	0.167	0.064
(95% CI)	(66.5-89.5)	(64.2-87.8)	(64.2-87.8)	(46.4-73.6)	(38.2-65.8)	(61.8-86.2)	(57.3-82.7)						
Specificity	94.5%	96.5%	92.0%	89.0%	92.5%	93.5%	93.0%	0.481	0.383	0.043	0.541	0.136	1.000
(95% CI)	(91.3-97.7)	(94.0-99.0)	(88.2-95.8)	(84.7-93.3)	(88.8-96.2)	(90.1-96.9)	(89.5-96.5)						
	**VK vs. BK + FK + AK + NIK**												
Accuracy	91.2%	86.0%	89.6%	78.4%	74.4%	87.2%	86.4%	0.066	0.597	<0.001	<0.001	0.006	<0.001
(95% CI)	(87.7-94.7)	(81.7-90.3)	(85.8-93.4)	(73.3-83.5)	(69.0-79.8)	(83.1-91.3)	(82.2-90.6)						
Sensitivity	80.0%	78.0%	76.0%	66.0%	62.0%	76.0%	72.0%	1.000	0.754	<0.143	0.064	0.359	0.302
(95% CI)	(68.9-91.1)	(66.5-89.5)	(64.2-87.8)	(52.9-79.1)	(48.5-75.5)	(64.2-87.8)	(59.6-84.4)						
Specificity	94.0%	88.0%	93.0%	81.5%	77.5%	90.0%	90.0%	0.029	0.832	<0.001	<0.001	0.012	<0.001
(95% CI)	(90.7-97.3)	(83.5-92.5)	(89.5-96.5)	(76.1-86.9)	(71.7-83.3)	(85.8-94.2)	(85.8-94.2)						
	**AK vs. BK + FK + VK + NIK**												

Accuracy	92.0%	93.2%	92.0%	89.2%	86.4%	90.8%	90.0%	0.607	1.000	0.230	0.013	0.541	0.108
(95% CI)	(88.6-95.4)	(90.1-96.3)	(88.6-95.4)	(85.4-93.0)	(82.2-90.6)	(87.2-94.4)	(86.3-93.7)						
Sensitivity	66.0%	72.0%	62.0%	46.0%	38.0%	62.0%	58.0%	0.549	0.815	0.052	0.007	0.115	0.041
(95% CI)	(52.9-79.1)	(59.6-84.4)	(48.5-75.5)	(32.2-59.8)	(24.5-51.5)	(48.5-75.5)	(44.3-71.7)						
Specificity	98.5%	98.5%	99.5%	100% (100-	98.5%	98.0%	98.0%	1.000	0.625	0.250	1.000	0.125	1.000
(95% CI)	(96.8-100)	(96.8-100)	(98.5-100)	100)	(96.8-100)	(96.1-99.9)	(96.1-99.9)						
	**NIK vs. BK + FK + VK + AK**												
Accuracy	92.8%	91.6%	90.4%	84.8%	84.4%	90.8%	92.0%	0.701	0.345	0.001	0.001	0.036	0.001
(95% CI)	(89.6-96.0)	(88.2-95.0)	(86.7-94.1)	(80.3-89.3)	(79.9-88.9)	(87.2-94.4)	(88.6-95.4)						
Sensitivity	84.0%	80.0%	90.0%	56.0%	72.0%	80.0%	82.0%	0.754	0.508	0.001	0.146	0.017	0.180
(95% CI)	(73.8-94.2)	(68.9-91.1)	(81.7-98.3)	(42.2-69.8)	(59.6-84.4)	(68.9-91.1)	(71.4-92.6)						
Specificity	95.0%	94.5%	90.5%	92.0%	87.5%	93.5%	94.5%	1.000	0.064	0.238	0.006	0.678	0.007
(95% CI)	(92.0-98.0)	(91.3-97.7)	(86.4-94.6)	(88.2-95.8)	(82.9-92.1)	(90.1-96.9)	(91.3-97.7)						

P1 indicates the p-value calculated between DeepIK and senior1 using the McNemar test. *P2* indicates the p-value calculated between DeepIK and senior2 using the McNemar test. *P3* indicates the p-value calculated between DeepIK and junior1 using the McNemar test. *P4* indicates the p-value calculated between DeepIK and junior2 using the McNemar test. *P5* indicates the p-value calculated between the junior1 and “junior1 + DeepIK” using the McNemar test. *P6* indicates the p-value calculated between the junior2 and “junior2 + DeepIK” using the McNemar test. BK, bacterial keratitis. FK, fungal keratitis. VK, viral keratitis. AK, amebic keratitis. NIK, noninfectious keratitis. CI, confidence interval.

Supplementary Table 17. Discrepancies between DeepIK and the ophthalmologists in the contest dataset.

**Keratitis type**	**DeepIK correctly diagnosed, all the ophthalmologists misdiagnosed**	**All the ophthalmologists correctly diagnosed, DeepIK misdiagnosed**
Bacterial keratitis	2	2
Fungal keratitis	1	2
Viral keratitis	0	2
Amebic keratitis	2	4
Noninfectious keratitis	0	1

Supplementary Table 18. Details of the trained CNN models

Model	Model size	Batch size	No. of epochs	Trainable parameters	Training Time	Testing Time
DeepIK	84.4MB	32	80	9.0e+06	2.15h	0.034s
DenseNet121	56.4MB	32	80	7.9e+06	2.13h	0.034s
InceptionResNetV2	415.28MB	32	80	5.6e+07	3.02h	0.036s
Swin-Transformer	663.56MB	32	80	8.8e+07	2.57h	0.033s

The models were trained and tested with GPU: GeForce RTX 2080Ti×4, CPU: Intel Xeon Gold 5220. Training time indicates the running time of each model in the whole training process. Testing time indicates the average time that the model needs to test every image. CNN, convolutional neural network. MB = Mbyte.

Supplementary Table 19. List of digital slit-lamp cameras used in each clinical center.

Clinical center	Location (City, Province)	Camera brand	Model	File type	File size per image
EHWMU	Wenzhou, Zhejiang	Sanyo	VPC-MZ3GX	JPG and BMP	1.54 to 6.25 MB
NEH	Ningbo, Zhejiang	Canton Optics	LS-7	JPG	3.73 to 6.42 MB
DNPH	Dalian, Liaoning	Haag-Streit	BQ 900	TIF	5.49 MB
FAHFMU	Fuzhou, Fujian	Kanghua	SLM-8E	JPG	1.34 to 2.87 MB
SHLU	Lanzhou, Gansu	Haag-Streit	BQ 900	JPG	1.23 to 1.61 MB
AEHNU	Nanchang, Jiangxi	Kanghua	SLM-8E	JPG	0.38 to 1.84 MB
AEHNMU	Nanjing, Jiangsu	Kanghua	SLM-7E	PNG	0.56 to 3.85 MB
PHNHAR	Yinchuan, Ningxia	Zeiss	SL120+Canon600D	JPG	0.10 to 0.58 MB
RHWU	Wuhan, Hubei	Sunkingdom	LS-6	JPG	0.91 to 2.08 MB
XNH	Xi'an, Shaanxi	Topcon	SL-D701	JPG	0.28 to 2.01 MB
TXHCSU	Changsha, Hunan	Haag-Streit	BQ 900	JPG	0.16 to 0.36 MB
AHGMU	Guiyang, Guizhou	Kanghua	SLM-KD4	JPG	2.13 to 10.40 MB

EHWMU, Eye Hospital of Wenzhou Medical University. NEH, Ningbo Eye Hospital. DNPH, Dalian No.3 People's Hospital. FAHFMU, First Affiliated Hospital of Fujian Medical University. SHLU, Second Hospital of Lanzhou University. AEHNU, Affiliated Eye Hospital of Nanchang University. AEHNMU, Affiliated Eye Hospital of Nanjing Medical University. PHNHAR, People's Hospital of Ningxia Hui Autonomous Region. RHWU, Renmin Hospital of Wuhan University. XNH, Xi'an No.1 Hospital. TXHCSU, Third Xiangya Hospital of Central South University. AHGMU, Affiliated Hospital of Guizhou Medical University.

Supplementary Table 20. Operations involved in data augmentation.

**Operation**	**Parameters**
Random horizontal and vertical flips	P=0.5
Random rotations	90, 180, 270 degrees
Random crop	224×224
Random contrast	Limit = 0.2
Random brightness	Limit = 0.2
