# Supplementary material for: Deep learning for multi-type infectious keratitis diagnosis: A nationwide, cross-sectional, multicenter study
Source: NPJ Digit Med. 2024 Jul 6;7:181. doi: 10.1038/s41746-024-01174-w (PMC11227533; doi:10.1038/s41746-024-01174-w)
Supplement: Supplementary file 2 — Reporting Summary [file 41746_2024_1174_MOESM2_ESM.pdf]

Corresponding author(s): Wei Chen

Last updated by author(s): Jun 17, 2024

Reporting Summary

Nature Portfolio wishes to improve the reproducibility of the work that we publish. This form provides structure for consistency and transparency in reporting. For further information on Nature Portfolio policies, see our Editorial Policies and the Editorial Policy Checklist.

Statistics

For all statistical analyses, confirm that the following items are present in the figure legend, table legend, main text, or Methods section.

n/a	Confirmed	
☐	☒	The exact sample size (*n*) for each experimental group/condition, given as a discrete number and unit of measurement
☐	☒	A statement on whether measurements were taken from distinct samples or whether the same sample was measured repeatedly
☐	☒	The statistical test(s) used AND whether they are one- or two-sided<br>*Only common tests should be described solely by name; describe more complex techniques in the Methods section.*
☒	☐	A description of all covariates tested
☒	☐	A description of any assumptions or corrections, such as tests of normality and adjustment for multiple comparisons
☐	☒	A full description of the statistical parameters including central tendency (e.g. means) or other basic estimates (e.g. regression coefficient) AND variation (e.g. standard deviation) or associated estimates of uncertainty (e.g. confidence intervals)
☐	☒	For null hypothesis testing, the test statistic (e.g. *F*, *t*, *r*) with confidence intervals, effect sizes, degrees of freedom and *P* value noted<br>*Give P values as exact values whenever suitable.*
☒	☐	For Bayesian analysis, information on the choice of priors and Markov chain Monte Carlo settings
☒	☐	For hierarchical and complex designs, identification of the appropriate level for tests and full reporting of outcomes
☐	☒	Estimates of effect sizes (e.g. Cohen's *d*, Pearson's *r*), indicating how they were calculated

Our web collection on statistics for biologists contains articles on many of the points above.

Software and code

Policy information about availability of computer code

Data collection: No software was used for data collection.

Data analysis: The two-sided 95% CIs for accuracy, sensitivity, and specificity were determined using the Wilson Score method through the Statsmodels package (version 0.11.1). For calculating the 95% CI of AUC, the Empirical Bootstrap method with 1,000 random replicates was employed. ROC curves were constructed utilizing the Matplotlib (version 3.3.1) and Scikit-learn (version 0.23.2) packages. Confusion matrices were leveraged to illustrate the classification results. The concordance between the outputs of the deep learning system and the reference standard was evaluated using unweighted Cohen kappa scores. We utilized a McNemar test to compare the accuracies, sensitivities, and specificities between the system and the ophthalmologists. Statistical analysis in this study was performed using Python 3.7.8 (Wilmington, Delaware, USA), with statistical significance set at a two-sided P-value below 0.05.

For manuscripts utilizing custom algorithms or software that are central to the research but not yet described in published literature, software must be made available to editors and reviewers. We strongly encourage code deposition in a community repository (e.g. GitHub). See the Nature Portfolio guidelines for submitting code & software for further information.

Data

Policy information about availability of data

All manuscripts must include a data availability statement. This statement should provide the following information, where applicable:

- Accession codes, unique identifiers, or web links for publicly available datasets
- A description of any restrictions on data availability
- For clinical datasets or third party data, please ensure that the statement adheres to our policy

The data substantiating the main findings of this study can be found in the manuscript and its Supplementary Information. Due to regulations imposed by hospitals and concerns regarding patient privacy, the raw datasets from individual clinical centers cannot be provided. Anonymized data is accessible for research purposes and can be obtained from the corresponding authors upon a reasonable request. The custom codes for DeepIK development and evaluation in this study are accessible on GitHub (https://github.com/jiangjiewei/DeepIK).

Research involving human participants, their data, or biological material

Policy information about studies with human participants or human data. See also policy information about sex, gender (identity/presentation), and sexual orientation and race, ethnicity and racism.

Reporting on sex and gender	The patient cohort had a mean age of 53.6 years (with a range of 0.25 to 100 years) and represented 41.8% women or girls.
Reporting on race, ethnicity, or other socially relevant groupings	All data were obtained from Chinese patients.
Population characteristics	The images were derived from 10,369 patients across 12 independent clinical centers across China. The patient cohort had a mean age of 53.6 years (with a range of 0.25 to 100 years) and represented 41.8% women or girls.
Recruitment	In total, 10,592 slit-lamp images (JPG format) acquired from 6,300 patients at EHWMU between May 2007 and October 2021 were utilized to develop a deep learning system. External test datasets including 7,206 slit-lamp images (JPG, TIF, PNG, and BMP format) obtained from 11 other clinical centers nationwide were employed to further evaluate the efficacy of the system. The development and external test datasets were annotated retrospectively, which may have introduced a certain level of selection bias but the prospective test indicated that this limitation may not be prominent.
Ethics oversight	This study adhered to the principles of the Declaration of Helsinki and received approval from both the institutional review boards/ethics committees of Ningbo Eye Hospital (NEH) (identifier, 2021-xjx-009) and the National Clinical Research Center for Ocular Diseases (NCRCOD) (identifier, 2022-222-K-176-01).

Note that full information on the approval of the study protocol must also be provided in the manuscript.

Field-specific reporting

Please select the one below that is the best fit for your research. If you are not sure, read the appropriate sections before making your selection.

☒ Life sciences ☐ Behavioural & social sciences ☐ Ecological, evolutionary & environmental sciences

For a reference copy of the document with all sections, see nature.com/documents/nr-reporting-summary-flat.pdf

Life sciences study design

All studies must disclose on these points even when the disclosure is negative.

Sample size	A total of 23,055 qualified images were utilized to construct and evaluate a deep learning system. The included images were derived from 10,369 patients across 12 independent clinical centers spanning the entire country. Specifically, the datasets consisted of 3,394 images of bacterial keratitis, 4,328 images of fungal keratitis, 8,224 images of viral keratitis, 446 images of amebic keratitis, and 6,663 images of noninfectious keratitis.
Data exclusions	Poor-quality images and images without sufficient diagnostic certainty were excluded from the study. In total, we excluded 1,452 poor-quality images and 3,624 images without sufficient diagnostic certainty.
Replication	PyTorch was utilized as the backend framework for training all the algorithms, which were executed on four Nvidia 2080TI graphics processing units (GPUs). To train the models, a mini-batch size of 32 was assigned for each GPU, resulting in 128 images processed in each iteration. The trainable parameters were updated based on the mean value computed from these samples. The training process made use of the adaptive moment estimation (ADAM) optimizer, with weight decay, β1, and β2 set to 1e-4, 0.9, and 0.999, respectively. The initial learning rate was set to 0.001 and was subsequently decreased by a factor of one-tenth every 20 epochs. Each model underwent training for 80 epochs, with the loss and accuracy being assessed on a validation dataset at each epoch to monitor the model's performance. The model exhibiting the highest accuracy on the validation dataset was ultimately applied to an internal test dataset.
Randomization	The development dataset was randomly divided into training, validation, and internal test datasets, with proportions of 70%, 15%, and 15%

Randomization	respectively. All images associated with the same patient should fall into one split to prevent data leakage and biased evaluation of performance.
Blinding	To mitigate bias from the competition, the ophthalmologists were not informed that they were in competition with DeepIK.

Reporting for specific materials, systems and methods

We require information from authors about some types of materials, experimental systems and methods used in many studies. Here, indicate whether each material, system or method listed is relevant to your study. If you are not sure if a list item applies to your research, read the appropriate section before selecting a response.

Materials & experimental systems

n/a	Involved in the study
☒	☐ Antibodies
☒	☐ Eukaryotic cell lines
☒	☐ Palaeontology and archaeology
☒	☐ Animals and other organisms
☐	☒ Clinical data
☒	☐ Dual use research of concern
☒	☐ Plants

Methods

n/a	Involved in the study
☒	☐ ChIP-seq
☒	☐ Flow cytometry
☒	☐ MRI-based neuroimaging

Clinical data

Policy information about clinical studies

All manuscripts should comply with the ICMJE guidelines for publication of clinical research and a completed CONSORT checklist must be included with all submissions.

Clinical trial registration	The study was registered at ClinicalTrials.gov (NCT05538793).
Study protocol	The study protocol was described in the Methods section.
Data collection	In total, 10,592 slit-lamp images (JPG format) acquired from 6,300 patients at EHWMU between May 2007 and October 2021 were utilized to develop a deep learning system. External test datasets including 7,206 slit-lamp images (JPG, TIF, PNG, and BMP format) obtained from 11 other clinical centers nationwide were employed to further evaluate the efficacy of the system. The prospective pilot study was conducted in EHWMU (Wenzhou from November 2021 to October 2022).
Outcomes	The primary outcome is the AUC of deep learning models. The secondary outcomes include accuracy, sensitivity, and specificity.

Plants

Seed stocks	NA
Novel plant genotypes	NA
Authentication	NA
